# Supplementary material for: Comparative genome analysis of Pasteurella multocida from Australian domestic animals suggests broad patterns of transmissions across multiple hosts and origins
Source: PLoS One. 2025 Aug 6;20(8):e0329807. doi: 10.1371/journal.pone.0329807 (PMC12327604; doi:10.1371/journal.pone.0329807)
Supplement: S1 Table — (PDF) [file pone.0329807.s001.pdf]

S1 Table. Details of *P. multocida* isolates from this study.

Isolate	Year	State	Host species	Isolation source	Host tissue	Disease	Capsular type	LPS type	*tox A*	Subsp.	Plasmid U51470	MH ST	RIRDC ST
CM2006-0650-0	2006	New South Wales	*Gallus gallus*	Commercial poultry farm	unknown	pasteurellosis	A	3	N	*septica*	N	338	183
CM2007-0542-0	2007	New South Wales	*Gallus gallus*	Commercial poultry farm	systemic	pasteurellosis	A	3	N	*septica*	N	37	23
CM2009-0453-0	2009	Western Australia	*Gallus gallus*	Commercial poultry farm	enteric	pasteurellosis	A	1	N	*septica*	N	38	171
CM2009-0556-0	2009	Victoria	*Canis lupus familiaris*	Domestic pet	skin	pasteurellosis	A	1	N	*septica*	N	-	37
CM2009-0682-0	2009	Victoria	*Felis catus*	Domestic pet	urine	pasteurellosis	A	1	N	*septica*	N	262	36
CM2009-0827-0	2009	Victoria	*Felis catus*	Domestic pet	urine	pasteurellosis	A	4	N	*septica*	N	-	527
CM2010-0113-1	2010	Victoria	*Felis catus*	Domestic pet	respiratory	pasteurellosis	A	5	N		N	-	-
CM2010-0286-0	2010	Victoria	*Sus scrofa domesticus*	N/A	skin	pasteurellosis	A	3	N		N	160	382
CM2010-0516-0	2010	Victoria	*Gallus gallus*	Commercial poultry farm	reproductive	pasteurellosis	A	1	N		N	-	30
CM2010-0545-2	2010	Victoria	*Felis catus*	Domestic pet	systemic	pasteurellosis	-	-	N	*septica*	N	-	-
CM2011-0151-1	2011	Victoria	*Canis lupus familiaris*	Domestic pet	skin	pasteurellosis	A	3	N	*septica*	N	338	183
CM2011-0458-1	2011	Victoria	*Dasyurus sp.*	Captive wildlife	lymphatic	pasteurellosis	-	6	N		N	-	-
CM2011-0584-0	2011	Victoria	*Canis lupus familiaris*	Domestic pet	skin	pasteurellosis	A	1	N	*septica*	N	38	171
CM2011-0629-0	2011	Victoria	*Dasyurus sp.*	Captive wildlife	respiratory	pasteurellosis	-	1	N		N	-	-
CM2013-0017-0	2013	Victoria	*Oryctolagus cuniculus*	Domestic pet	skin	pasteurellosis	A	3	N	*septica*	N	-	451
CM2013-0823-1	2013	Victoria	*Anas domesticus*	Commercial farm	musculoskeletal	pasteurellosis	A	3	N	*septica*	N	37	23
CM2014-0491-0	2014	Victoria	*Felis catus*	Domestic pet	skin	pasteurellosis	A	1	N		N	-	-
CM2014-0657-0	2014	Victoria	*Oryctolagus cuniculus*	Domestic pet	systemic	pasteurellosis	A	4	N	*septica*	N	-	527

CM2015-0068-1	2015	Victoria	*Ovis aries*	Commercial farm	systemic	pasteurellosis	A	3	P		N	65	132
CM2015-0090-1	2015	Victoria	*Ovis aries*	Commercial farm	respiratory	pasteurellosis	A	6	N		N	162	12
CM2015-0350-0	2015	Victoria	*Oryctolagus cuniculus*	Domestic pet	systemic	pasteurellosis	A	-	N	*septica*	P	-	-
CM2016-1071-0	2016	Victoria	*Bos taurus*	Commercial farm	respiratory	pasteurellosis	A	3	N		N	1	79
CM2016-1071-1	2016	Victoria	*Bos taurus*	N/A	respiratory	pasteurellosis	A	3	N		N	1	79
CM2017-0508-0	2017	Victoria	*Environment*	Veterinary hospital	N/A	N/A	A	3	N	*septica*	N	-	3
CM2017-0740-5	2017	Victoria	*Gallus gallus*	N/A	systemic	pasteurellosis	A	3	N		P	124	8
CM2018-0104-0	2018	Victoria	*Gallus gallus*	Commercial farm	systemic	pasteurellosis	A	3	N		N	124	8
CM2018-0111-0	2018	Victoria	*Gallus gallus*	Commercial farm	systemic	pasteurellosis	A	3	N		N	124	8
CM2018-0111-1	2018	Victoria	*Gallus gallus*	Commercial farm	systemic	pasteurellosis	A	3	N		N	124	8
CM2018-0364-0	2018	Victoria	*Felis catus*	Domestic pet	ear	pasteurellosis	A	-	N		N	-	214
CM2018-0476-1	2018	Victoria	*Canis lupus familiaris*	Domestic pet	ear	pasteurellosis	A	3	N	*septica*	N	87	359
CM2018-0643-0	2018	Victoria	*Felis catus*	Domestic pet	skin	pasteurellosis	A	4	N	*septica*	N	324	527
CM2019-0106-0	2019	Victoria	*Ovis aries*	Commercial farm	respiratory	pasteurellosis	A	1	P		N	-	-
CM2019-0177-0	2019	Victoria	*Canis lupus familiaris*	Domestic pet	unknown	pasteurellosis	A	3	N		N	-	-
CM2019-0327-0	2019	Victoria	*Ovis aries*	Commercial farm	respiratory	pasteurellosis	A	3	P		N	65	132
CM2019-0508-0	2019	Victoria	*Felis catus*	Domestic pet	systemic	pasteurellosis	A	3	N	*septica*	N	33	37
CM2019-0543-0	2019	Victoria	*Dasyurus sp.*	Captive wildlife	skin	pasteurellosis	A	1	N		N	-	-
CM2019-0603-0	2019	Victoria	*Felis catus*	Domestic pet	respiratory	pasteurellosis	-	2	N	*septica*	N	324	527
CM2019-0691-0	2019	Queensland	*Gallus gallus*	Commercial poultry farm	systemic	pasteurellosis	A	4	N		P	151	9

CM2019-0691-1	2019	Queensland	*Gallus gallus*	Commercial poultry farm	systemic	pasteurellosis	A	3	N		P	151	9
CM2019-0692-0	2019	Queensland	*Gallus gallus*	Commercial poultry farm	systemic	pasteurellosis	A	3	N		P	151	9
CM2019-0891-0	2019	Victoria	*Felis catus*	Domestic pet	skin	pasteurellosis	A	3	N	*septica*	N	-	3
CM2020-0075-0	2020	Victoria	*Canis lupus familiaris*	Domestic pet	skin	pasteurellosis	A	3	N	*septica*	N	38	171
CM2020-1034-0	2020	Victoria	*Leptailurus serval*	Captive wildlife	respiratory	pasteurellosis	A	1	N		N	-	-
CM2020-1144-0	2020	Victoria	*Felis catus*	Domestic pet	urine	pasteurellosis	A	3	N	*septica*	N	-	451
CM2021-0098-0	2021	Victoria	*Felis catus*	Domestic pet	skin	pasteurellosis	A	3	N	*septica*	N	324	527
CM2021-0114-0	2021	Victoria	*Felis catus*	Domestic pet	urine	pasteurellosis	A	4	N	*septica*	N	227	25
CM2021-0118-0	2021	Victoria	*Felis catus*	Domestic pet	respiratory	pasteurellosis	A	3	N	*septica*	N	-	451
CM2022-0178-1	2022	Victoria	*Oryctolagus cuniculus*	Domestic pet	ear	pasteurellosis	A	3	N		N	227	25
CM2022-0178-2	2022	Victoria	*Oryctolagus cuniculus*	Domestic pet	ear	pasteurellosis	A	3	N		P	227	25
CM2022-0184-0	2022	Victoria	*Gallus gallus*	Commercial free range layer farm	respiratory	pasteurellosis	A	3	N		N	124	8
CM2022-0265-1	2022	Victoria	*Gallus gallus*	Commercial free range layer farm	respiratory	pasteurellosis	A	3	N		N	151	9
CM2022-0265-2	2022	Victoria	*Gallus gallus*	Commercial free range layer farm	respiratory	pasteurellosis	A	3	N		N	151	9
CM2022-0430-0	2022	Victoria	*Gallus gallus*	Commercial poultry farm	respiratory	pasteurellosis	A	3	N		N	159	20
CM2023-0122-0	2023	Victoria	*Bos taurus*	N/A	respiratory	pasteurellosis	A	3	N		N	159	394
CM2023-0137-0	2023	Victoria	*Gallus gallus*	Commercial poultry farm	systemic	pasteurellosis	A	-	N		N	159	20
CM2023-0222-2	2023	Victoria	*Bos taurus*	N/A	systemic	pasteurellosis	A	3	N		N	159	394

CM2023-0226-0	2023	Victoria	*Gallus gallus*	Commercial poultry farm	systemic	pasteurellosis	A	3	N	*septica*	N	214	169
CM2023-0265-0	2023	Victoria	*Bos taurus*	Commercial dairy farm	systemic	pasteurellosis	A	3	N		N	1	79
CM2023-0277-0	2023	Victoria	*Bos taurus*	Commercial dairy farm	systemic	pasteurellosis	A	3	N		N	6	123
