# Supplementary material for: Comparative genome analysis of Pasteurella multocida from Australian domestic animals suggests broad patterns of transmissions across multiple hosts and origins
Source: PLoS One. 2025 Aug 6;20(8):e0329807. doi: 10.1371/journal.pone.0329807 (PMC12327604; doi:10.1371/journal.pone.0329807)
Supplement: S2 Table — (PDF) [file pone.0329807.s002.pdf]

S2 Table. Details of the published *P. multocida* genomes used in comparative analyses within this study.

Accession	Strain	Year	Host category	Host	Country	Capsular type	LPS type	ToxA	MH_ST	RIRDC_ST
GCF_000006825.1	Pm70	-	-	-	-	CapACapF	LpsL3	-	9	25
GCF_000234745.1	36950	-	-	-	-	CapA	LpsL3	-	79	1
GCF_000255915.1	HN06	-	-	-	-	CapD	LpsL6	toxA	50	11
GCF_000259545.1	3480	-	Pig	Porcine	-	CapA	LpsL6	-	74	10
GCF_000291605.1	P52VAC	2011	Ruminant	Bovine	India	-	LpsL2	-	122	44
GCF_000296345.2	VTCCBAA264	2011	Ruminant	Bovine	India	CapB	-	-	122	44
GCF_000298655.1	X73	-	-	-	-	CapA	LpsL1	-	60	94
GCF_000298675.1	P1059	1962	Bird	Turkey	USA	CapA	LpsL3	-	8	124
GCF_000409915.1	1500E	2000	Ruminant	Bovine	United_Kingdom	CapA	LpsL3	-	79	1
GCF_000412015.1	P1933	2004	Ruminant	Bovine	United_Kingdom	CapA	LpsL3	-	80	3
GCF_000412035.1	2000	2000	Ruminant	Bovine	United_Kingdom	CapA	LpsL3	-	79	1
GCF_000412075.1	671/90	1990	Ruminant	Bovine	United_Kingdom	CapA	LpsL6	-	349	7
GCF_000412105.1	RIIF	1999	Ruminant	Ovine	United_Kingdom	CapACapF	LpsL3	-	339	17
GCF_000412125.1	1500C	2000	Ruminant	Bovine	United_Kingdom	CapB	-	-	122	44
GCF_000413135.1	P1062	1962	Ruminant	Bovine	USA	CapA	LpsL3	-	80	3
GCF_000469095.1	PMTB	2003	-	-	Malaysia	CapB	LpsL2	-	-	44
GCF_000478235.1	93002	1995	Ruminant	Bovine	Sri_Lanka	-	LpsL3	-	79	1
GCF_000512395.1	HB03	-	Pig	Porcine	-	CapA	LpsL3	-	13	3
GCF_000731735.1	2213	2012	Ruminant	Bovine	India	CapB	LpsL2	-	122	44
GCF_000731745.1	3213	2012	Ruminant	Bovine	India	CapB	LpsL2	-	122	44
GCF_000754275.1	ATCC_43137	-	-	-	-	CapA	LpsL3	-	13	3
GCF_000973565.1	OH1905	-	-	-	-	CapA	LpsL4	-	31	221
GCF_001027665.1	PVAcc	2011	-	-	Pakistan	CapB	LpsL2	-	122	44
GCF_001027685.1	V1	2011	Ruminant	Bovine	Pakistan	CapB	LpsL2	-	122	44
GCF_001027695.1	TX1	2012	Ruminant	Bovine	Pakistan	CapB	LpsL2	-	122	44
GCF_001027735.1	Islm	2011	Ruminant	Bovine	Pakistan	CapB	LpsL2	-	122	44
GCF_001027755.1	THA	2006	Ruminant	Bovine	Thailand	CapB	LpsL2	-	122	44
GCF_001027805.1	Pesh	2011	Ruminant	Bovine	Pakistan	CapB	LpsL2	-	122	44
GCF_001027815.1	THD	2009	Ruminant	Bovine	Thailand	CapB	LpsL2	-	122	44
GCF_001027825.1	THF	2011	Ruminant	Bovine	Thailand	CapB	LpsL2	-	122	44
GCF_001028205.1	Faisal	2011	Ruminant	Bovine	Pakistan	CapB	LpsL2	-	122	44
GCF_001028225.1	ATTK	2010	Ruminant	Bovine	Pakistan	CapB	LpsL2	-	122	44
GCF_001028245.1	Karachi	2011	Ruminant	Bovine	Pakistan	CapB	LpsL2	-	122	44
GCF_001029495.1	BUKK	2008	Ruminant	Bovine	Pakistan	CapB	LpsL2	-	122	44
GCF_001510775.1	SMC1	2015	Human	Human	Malaysia	CapA	LpsL3	-	128	222

GCF_001578435.2	PMTB2.1	-	-	-	-	CapA	LpsL1	-	201	103
GCF_001661585.1	HB02	2010	Bird	Duck	China	CapA	LpsL1	-	129	128
GCF_001662525.1	C48-1	1953	Bird	Chicken	China	CapA	LpsL1	-	129	128
GCF_001663095.1	HB01	2010	-	-	China	CapA	LpsL3	-	79	1
GCF_001670425.2	HN141014	2014	Bird	Duck	China	CapA	LpsL1	-	129	128
GCF_001670435.1	ATCC_11039	1955	Bird	Chicken	-	CapA	LpsL1	-	60	94
GCF_001670445.1	DY120818	2012	Bird	Duck	China	CapA	LpsL1	-	129	128
GCF_001670455.1	RCAD0276	2015	Bird	Duck	China	CapA	LpsL1	-	129	128
GCF_001670525.1	ATCC_1662	1955	Bird	Turkey	-	CapA	LpsL3	-	53	223
GCF_001670535.1	ATCC_2095	1962	Bird	Turkey	-	CapA	LpsL5	-	159	130
GCF_001670545.1	ATCC_1702	1958	Bird	Turkey	-	CapA	LpsL2	-	156	224
GCF_001670555.1	ATCC_2100	1965	Bird	Turkey	-	CapA	LpsL6	-	27	10
GCF_001670775.1	ATCC_15742	1955	Bird	Turkey	-	CapA	LpsL3	-	8	124
GCF_001721885.1	Pm-3	2013	Ruminant	Bovine	China	CapA	LpsL3	-	79	1
GCF_001857595.1	HS_SKN01	2016	Ruminant	Bovine	India	CapB	LpsL2	-	122	44
GCF_001874105.1	unmsm	2007	Ruminant	Alpaca	Peru	CapA	LpsL6	toxA	321	229
GCF_001874445.1	Razi_Pm0001	1936	Ruminant	Bovine	Iran	CapB	LpsL2	-	122	44
GCF_001929465.1	2154PM	2011	Ruminant	Bovine	USA	CapA	LpsL3	-	79	1
GCF_001929525.1	2320PM	2011	Ruminant	Bovine	USA	CapA	LpsL3	-	79	1
GCF_001929565.1	2403PM	2011	Ruminant	Bovine	USA	CapA	LpsL3	-	79	1
GCF_001929595.1	2450PM	2011	Ruminant	Bovine	USA	CapA	LpsL3	-	79	1
GCF_001929655.1	2512PM	2011	Ruminant	Bovine	USA	CapA	LpsL3	-	79	1
GCF_001929705.1	2578PM	2011	Ruminant	Bovine	USA	CapA	LpsL3	-	79	1
GCF_001929765.1	2633PM	2011	Ruminant	Bovine	USA	CapA	LpsL3	-	79	1
GCF_001929785.1	2612PM	2011	Ruminant	Bovine	USA	CapA	LpsL3	-	79	1
GCF_001929855.1	2930PM	2011	Ruminant	Bovine	USA	CapA	LpsL3	-	79	1
GCF_001929905.1	2125PM	2011	Ruminant	Bovine	USA	CapA	LpsL3	-	79	1
GCF_001929985.1	2165PM	2011	Ruminant	Bovine	USA	CapA	LpsL3	-	79	1
GCF_001930065.1	2267PM	2011	Ruminant	Bovine	USA	CapA	LpsL3	-	79	1
GCF_001930115.1	2297PM	2011	Ruminant	Bovine	USA	CapA	LpsL3	-	79	1
GCF_001930285.1	2389PM	2011	Ruminant	Bovine	USA	CapA	LpsL3	-	79	1
GCF_001930305.1	2335PM	2011	Ruminant	Bovine	USA	CapA	LpsL3	-	79	1
GCF_001930385.1	2428PM	2011	Ruminant	Bovine	USA	CapA	LpsL3	-	79	1
GCF_001930405.1	2497PM	2011	Ruminant	Bovine	USA	CapA	LpsL3	-	79	1
GCF_001930445.1	2526PM	2011	Ruminant	Bovine	USA	CapA	LpsL3	-	79	1
GCF_001930465.1	2597PM	2011	Ruminant	Bovine	USA	CapA	LpsL3	-	79	1
GCF_001930525.1	2668PM	2011	Ruminant	Bovine	USA	CapA	LpsL3	-	79	1
GCF_001930605.1	2887PM	2011	Ruminant	Bovine	USA	CapA	LpsL3	-	79	1
GCF_001930705.1	3022PM	2011	Ruminant	Bovine	USA	CapA	LpsL3	-	79	1

GCF_001930765.1	3045PM	2011	Ruminant	Bovine	USA	CapA	LpsL3	-	79	1
GCF_001930875.1	3275PM	2011	Ruminant	Bovine	USA	CapA	LpsL3	-	79	1
GCF_001930895.1	3347PM	2011	Ruminant	Bovine	USA	CapA	LpsL3	-	79	1
GCF_001930925.1	3384PM	2011	Ruminant	Bovine	USA	CapA	LpsL3	-	79	1
GCF_001930965.1	3483PM	2011	Ruminant	Bovine	USA	CapA	LpsL3	-	79	1
GCF_001931115.1	68BPM	2014	Ruminant	Bovine	USA	CapA	LpsL3	-	79	1
GCF_001931145.1	69APM	2014	Ruminant	Bovine	USA	CapA	LpsL3	-	79	1
GCF_001931155.1	654BPM	2014	Ruminant	Bovine	USA	CapA	LpsL3	-	79	1
GCF_001931185.1	657BPM	2014	Ruminant	Bovine	USA	CapA	LpsL3	-	79	1
GCF_001931225.1	8522APM	2014	Ruminant	Bovine	USA	CapA	LpsL3	-	79	1
GCF_001931235.1	2901PM	2011	Ruminant	Bovine	USA	CapA	LpsL3	-	79	1
GCF_001931265.1	2969PM	2011	Ruminant	Bovine	USA	CapA	LpsL3	-	79	1
GCF_001931455.1	J1APM	2014	Ruminant	Bovine	USA	CapA	LpsL3	-	79	1
GCF_001956795.1	HN07	-	-	-	-	CapACapF	LpsL3	-	9	12
GCF_002023805.1	CIRMBP-0760	2003	Rabbit	Rabbit	France	CapA	LpsL6	-	298	225
GCF_002023825.1	CIRMBP-0758	2003	Rabbit	Rabbit	France	CapA	LpsL6	-	298	225
GCF_002023835.1	CIRMBP-0812	2007	Rabbit	Rabbit	France	CapA	LpsL3	-	204	12
GCF_002023845.1	CIRMBP-0749	2003	Rabbit	Rabbit	France	CapA	LpsL3	-	204	12
GCF_002023885.1	CIRMBP-0782	2006	Rabbit	Rabbit	France	CapACapF	LpsL3	-	9	12
GCF_002023905.1	CIRMBP-0827	-	Rabbit	Rabbit	France	CapA	LpsL3	-	204	-
GCF_002023925.1	CIRMBP-0877	-	Rabbit	Rabbit	France	CapD	LpsL6	-	50	11
GCF_002023935.1	CIRMBP-0817	2007	Rabbit	Rabbit	France	CapA	LpsL6	-	74	10
GCF_002023945.1	CIRMBP-0783	2006	Rabbit	Rabbit	France	CapA	LpsL3	-	204	12
GCF_002023985.1	CIRMBP-0922	-	Rabbit	Rabbit	France	CapACapF	LpsL3	-	9	12
GCF_002024005.1	CIRMBP-0835	-	Rabbit	Rabbit	France	CapA	LpsL3	-	9	12
GCF_002024015.1	CIRMBP-0872	-	Rabbit	Rabbit	France	CapACapF	LpsL3	-	9	12
GCF_002024045.1	CIRMBP-0906	-	Rabbit	Rabbit	France	CapACapF	LpsL3	-	9	12
GCF_002024065.1	CIRMBP-0747	2003	Rabbit	Rabbit	France	CapACapF	LpsL3	-	9	12
GCF_002024085.1	CIRMBP-0927	-	Rabbit	Rabbit	France	CapACapF	LpsL3	-	9	12
GCF_002059225.1	CIRMBP-0884	-	Rabbit	Rabbit	France	CapACapF	LpsL3	-	9	12
GCF_002068175.1	CIRMBP-0873	-	Rabbit	Rabbit	France	CapACapF	LpsL3	-	428	191
GCF_002073255.2	FDAARGOS_218	-	Environment	Environment	USA	CapA	LpsL3	-	8	124
GCF_002073295.2	FDAARGOS_216	1958	Bird	Chicken	USA	CapA	LpsL1	-	60	94
GCF_002083205.2	FDAARGOS_261	2014	Human	Human	USA	CapA	LpsL1	-	242	226
GCF_002083215.2	FDAARGOS_220	-	Environment	Environment	USA	CapA	LpsL2	-	156	224
GCF_002083265.2	FDAARGOS_217	-	Ruminant	Bovine	USA	CapB	LpsL2	-	122	64
GCF_002393385.1	FDAARGOS_384	2015	Human	Human	USA	CapA	LpsL3	-	25	227
GCF_002591295.1	FDAARGOS_385	2015	Human	Human	USA	CapA	LpsL3	-	25	227
GCF_002750755.1	SH04	2014	Pig	Porcine	China	CapA	LpsL6	-	456	228

GCF_002750815.1	SH03	2014	Pig	Porcine	China	CapA	LpsL6	-	74	-
GCF_002750835.1	SH02	2014	Pig	Porcine	China	CapD	LpsL6	-	457	11
GCF_002750875.1	SH05	2014	Pig	Porcine	China	CapACapF	LpsL3	-	9	12
GCF_002750885.1	SH06	2014	Pig	Porcine	China	CapA	LpsL6	-	74	10
GCF_002817545.1	SH01	2014	Pig	Porcine	China	CapD	LpsL6	-	457	11
GCF_002859245.1	USDA-ARS-USMARC-60494	2013	Ruminant	Bovine	USA	CapA	LpsL3	-	79	1
GCF_002859265.1	USDA-ARS-USMARC-60215	2013	Ruminant	Bovine	USA	CapA	LpsL3	-	79	1
GCF_002859285.1	USDA-ARS-USMARC-60717	2013	Ruminant	Bovine	USA	CapA	LpsL3	-	79	1
GCF_002859305.1	USDA-ARS-USMARC-60213	2013	Ruminant	Bovine	USA	CapA	LpsL3	-	79	1
GCF_002859325.1	USDA-ARS-USMARC-59910	2013	Ruminant	Bovine	USA	CapA	LpsL3	-	79	1
GCF_002859345.1	USDA-ARS-USMARC-60380	2013	Ruminant	Bovine	USA	CapA	LpsL3	-	79	1
GCF_002859365.1	USDA-ARS-USMARC-60713	2013	Ruminant	Bovine	USA	CapA	LpsL3	-	79	1
GCF_002859385.1	USDA-ARS-USMARC-60224	2013	Ruminant	Bovine	USA	CapA	LpsL3	-	79	1
GCF_002859405.1	USDA-ARS-USMARC-60712	2013	Ruminant	Bovine	USA	CapA	LpsL3	-	79	1
GCF_002859425.1	USDA-ARS-USMARC-60675	2013	Ruminant	Bovine	USA	CapA	LpsL3	-	79	1
GCF_002859445.1	USDA-ARS-USMARC-60248	2013	Ruminant	Bovine	USA	CapA	LpsL3	-	79	1
GCF_002859465.1	USDA-ARS-USMARC-60385	2013	Ruminant	Bovine	USA	CapA	LpsL3	-	80	3
GCF_002859485.1	USDA-ARS-USMARC-59962	2013	Ruminant	Bovine	USA	CapA	LpsL3	-	79	1
GCF_002859505.1	USDA-ARS-USMARC-60214	2013	Ruminant	Bovine	USA	CapA	LpsL3	-	-	-
GCF_002859525.1	USDA-ARS-USMARC-60381	2013	Ruminant	Bovine	USA	CapA	LpsL3	-	79	1
GCF_002859545.1	USDA-ARS-USMARC-60714	2013	Ruminant	Bovine	USA	CapA	LpsL3	-	79	1
GCF_002891825.2	CQ6	2013	Ruminant	Bovine	China	CapA	LpsL3	-	79	1
GCF_002891845.2	CQ2	2013	Ruminant	Bovine	China	CapA	LpsL3	-	79	1
GCF_002893985.1	B	2013	Ruminant	Bovine	China	CapB	LpsL2	-	122	44

GCF_002900115.1	UNMSM2	2013	Ruminant	Alpaca	Peru	CapA	LpsL6	toxA	321	229
GCF_002930755.1	S298D	2016	Dog/Cat	Canine	Greece	CapACapF	LpsL1	-	4	135
GCF_002930775.1	PY81579	2016	Human	Human	Greece	CapACapF	LpsL1	-	4	135
GCF_002948995.1	161215033201-1	2016	Human	Human	Netherlands	CapA	-	-	39	137
GCF_003061265.1	20N	2016	-	-	China	CapA	LpsL3	-	365	-
GCF_003061385.1	9N	2016	-	-	China	CapACapF	LpsL3	-	9	25
GCF_003261335.1	4407	2010	Ruminant	Bovine	-	CapA	LpsL6	-	206	7
GCF_003261435.1	3361	2006	Ruminant	Bovine	-	CapA	LpsL3	-	79	1
GCF_003261515.1	14424	2010	Ruminant	Bovine	-	CapA	LpsL3	-	79	1
GCF_003268295.1	BAUTB2	2016	Ruminant	Bovine	Bangladesh	CapB	LpsL2	-	122	44
GCF_003402875.1	PM8-1	2015	Pig	Porcine	China	CapA	LpsL3	-	13	3
GCF_003402895.1	TB168	2017	-	-	China	CapA	LpsL3	-	13	3
GCF_003402915.1	EB168	2017	-	-	China	CapA	LpsL3	-	13	3
GCF_003402935.1	BS168	2017	-	-	China	CapA	LpsL3	-	13	3
GCF_004114645.1	PM3	2015	Ruminant	Bovine	Pakistan	CapB	LpsL2	-	122	44
GCF_004286935.1	C51-2	1987	Rabbit	Rabbit	China	CapA	LpsL1	-	129	128
GCF_004286945.1	C48-1	1953	Bird	Chicken	China	CapA	LpsL1	-	129	128
GCF_004286955.1	C51-17	1989	Rabbit	Rabbit	China	CapA	LpsL1	-	129	128
GCF_004286965.1	Pm72-4	1972	Bird	Goose	China	CapA	LpsL1	-	-	128
GCF_004286975.1	Pm731	-	Bird	Goose	-	CapA	LpsL1	-	129	128
GCF_004287035.1	C51-3	1986	Rabbit	Rabbit	China	CapA	LpsL3	-	5	132
GCF_004287045.1	C44-1	1955	Pig	Porcine	China	CapB	LpsL2	-	122	73
GCF_004327765.1	232	2001	Ruminant	Bovine	USA	CapA	LpsL3	-	79	1
GCF_004336735.1	MSP58	2019	Dog/Cat	Feline	USA	CapACapF	LpsL3	-	9	25
GCF_004792555.1	FCf15	2004	Bird	Duck	China	CapA	LpsL1	-	129	207
GCF_004792575.1	FCf71	2013	Bird	Duck	China	CapA	LpsL1	-	129	230
GCF_004792595.1	FCf45	2009	Bird	Duck	China	CapA	LpsL1	-	342	231
GCF_004792615.1	FCf76	2015	Bird	Goose	China	CapA	LpsL1	-	129	128
GCF_004792635.1	FCf83	2015	Bird	Duck	China	CapA	LpsL1	-	129	230
GCF_006232125.1	HN01	2017	Ruminant	Caprine	China	CapD	LpsL3	toxA	320	181
GCF_006274235.1	EB104	2017	-	-	China	CapA	LpsL3	-	13	3
GCF_006351865.1	PM_8-6	2015	Pig	Porcine	China	CapA	LpsL3	-	13	3
GCF_008693845.1	FDAARGOS_644	-	Human	Human	USA	CapA	-	-	458	232
GCF_009646155.1	PM22	2011	Ruminant	Bovine	USA	CapA	LpsL3	-	79	1
GCF_009663815.1	P52	-	Ruminant	Bovine	India	CapB	LpsL2	-	122	44
GCF_009739555.1	Q	2010	Bird	Duck	China	CapA	LpsL1	-	129	128
GCF_009739575.1	CQ7	2013	Ruminant	Bovine	China	CapA	LpsL3	-	7	133
GCF_009901975.1	GH161213	2016	Bird	Duck	China	CapA	LpsL1	-	342	231
GCF_010443215.1	HS_Canada1	2018	Ruminant	Bovine	Canada	CapB	LpsL2	-	459	233

GCF_011149495.1	KVNON-213	2018	Dog/Cat	Feline	South_Korea	CapACapF	LpsL1	-	-	-
GCF_011390865.1	VP161	1995	Bird	Chicken	Viet_Nam	CapA	LpsL1	-	129	230
GCF_012126375.1	P-mult-10-KZ	2013	Ruminant	Bovine	Kazakhstan	CapB	LpsL2	-	122	64
GCF_012271895.1	P-mult-15-KZ	2010	Horse	Equine	Kazakhstan	CapB	LpsL2	-	460	64
GCF_012271915.1	P-mult-5-KZ	2006	Horse	Equine	Kazakhstan	CapB	LpsL2	-	460	64
GCF_012928515.1	Clemson	1967	Bird	Chicken	USA	CapA	LpsL3	-	235	234
GCF_012952165.1	92_67_2	1992	Bird	Chicken	USA	CapA	LpsL3	-	8	124
GCF_012952185.1	92_2667	1992	Bird	Chicken	USA	CapA	LpsL3	-	461	235
GCF_012952205.1	86_1913	1986	Bird	Turkey	USA	CapA	LpsL3	-	463	236
GCF_012952225.1	93_182	1993	Bird	Chicken	USA	CapA	LpsL3	-	8	124
GCF_013012025.1	HNA16	2017	Pig	Porcine	China	CapA	LpsL6	-	-	-
GCF_013012805.1	HN05	2017	Pig	Porcine	China	CapD	LpsL6	-	50	11
GCF_013012825.1	HN04	2017	Pig	Porcine	China	CapB	LpsL2	-	122	237
GCF_013012845.1	HNA01	2017	Pig	Porcine	China	CapA	LpsL3	-	7	133
GCF_013012855.1	HNA04	2017	Pig	Porcine	China	CapA	LpsL6	-	74	10
GCF_013012885.1	HNA03	2017	Pig	Porcine	China	CapA	LpsL3	-	13	3
GCF_013012895.1	HNA02	2017	Pig	Porcine	China	CapA	LpsL6	-	464	10
GCF_013012905.1	HNA05	2017	Pig	Porcine	China	CapA	LpsL6	-	74	10
GCF_013012945.1	HNA07	2017	Pig	Porcine	China	CapA	LpsL6	-	74	10
GCF_013012965.1	HNA06	2017	Pig	Porcine	China	CapA	LpsL6	-	27	10
GCF_013012975.1	HNA09	2017	Pig	Porcine	China	CapA	LpsL3	-	13	3
GCF_013012985.1	HNA10	2017	Pig	Porcine	China	CapA	LpsL6	-	74	10
GCF_013013025.1	HNA08	2017	Pig	Porcine	China	CapA	LpsL3	-	465	3
GCF_013013045.1	HNA11	2017	Pig	Porcine	China	CapA	LpsL6	-	74	10
GCF_013013065.1	HNA12	2017	Pig	Porcine	China	CapA	LpsL6	-	74	10
GCF_013013075.1	HNA14	2017	Pig	Porcine	China	CapA	LpsL3	-	13	3
GCF_013013085.1	HNA13	2017	Pig	Porcine	China	CapA	LpsL3	-	13	3
GCF_013013125.1	HNA15	2017	Pig	Porcine	China	CapA	LpsL3	-	13	3
GCF_013013135.1	HNA17	2017	Pig	Porcine	China	CapA	LpsL3	-	466	3
GCF_013013165.1	HNA19	2017	Pig	Porcine	China	CapA	LpsL3	-	13	3
GCF_013013175.1	HNA18	2017	Pig	Porcine	China	CapA	LpsL3	-	13	3
GCF_013013205.1	HNA20	2017	Pig	Porcine	China	CapA	LpsL3	-	13	3
GCF_013013215.1	HNA22	2017	Pig	Porcine	China	CapA	LpsL6	-	74	172
GCF_013013225.1	HNA21	2017	Pig	Porcine	China	CapA	LpsL6	-	74	172
GCF_013013235.1	HND01	2017	Pig	Porcine	China	CapD	LpsL6	-	50	11
GCF_013013245.1	HND02	2017	Pig	Porcine	China	CapD	LpsL6	-	287	134
GCF_013013305.1	HND07	2017	Pig	Porcine	China	CapD	LpsL6	-	50	11
GCF_013013315.1	HND04	2017	Pig	Porcine	China	CapD	LpsL6	-	467	146
GCF_013013345.1	HND06	2017	Pig	Porcine	China	CapD	LpsL6	-	50	11

GCF_013013355.1	HND03	2017	Pig	Porcine	China	CapD	LpsL6	-	50	11
GCF_013013365.1	HND05	2017	Pig	Porcine	China	CapD	LpsL6	-	468	11
GCF_013013405.1	HND09	2017	Pig	Porcine	China	CapD	LpsL6	-	50	11
GCF_013013425.1	HND12	2017	Pig	Porcine	China	CapD	LpsL6	-	287	134
GCF_013013435.1	HND08	2017	Pig	Porcine	China	CapD	LpsL6	-	50	11
GCF_013013455.1	HND10	2017	Pig	Porcine	China	CapD	LpsL6	-	50	11
GCF_013013475.1	HND11	2017	Pig	Porcine	China	CapD	LpsL6	-	457	11
GCF_013013505.1	HND13	2017	Pig	Porcine	China	CapD	LpsL6	-	287	134
GCF_013013525.1	HND15	2017	Pig	Porcine	China	CapD	LpsL6	-	50	11
GCF_013013535.1	HND16	2017	Pig	Porcine	China	CapD	LpsL6	-	469	11
GCF_013013545.1	HND14	2017	Pig	Porcine	China	CapD	LpsL6	-	50	11
GCF_013013565.1	HND17	2017	Pig	Porcine	China	CapD	LpsL6	-	50	11
GCF_013013585.1	HND18	2017	Pig	Porcine	China	CapD	LpsL6	-	470	11
GCF_013013625.1	HND19	2017	Pig	Porcine	China	CapD	LpsL6	-	50	11
GCF_013013645.1	HND20	2017	Pig	Porcine	China	CapD	LpsL6	-	50	11
GCF_013013655.1	HNF02	2017	Pig	Porcine	China	CapACapF	LpsL3	-	9	12
GCF_013013675.1	HND21	2017	Pig	Porcine	China	CapD	LpsL6	-	50	11
GCF_013013685.1	HNF01	2017	Pig	Porcine	China	CapACapF	LpsL3	-	9	12
GCF_014058465.1	A0419	1990	Ruminant	Bovine	United_Kingdom	CapA	LpsL6	-	349	7
GCF_014058485.1	A0757	1990	Ruminant	Bovine	United_Kingdom	CapA	LpsL6	-	349	7
GCF_014058585.1	X0120	1990	Ruminant	Bovine	United_Kingdom	CapA	LpsL6	-	349	7
GCF_014058605.1	X1053	1990	Ruminant	Bovine	United_Kingdom	CapA	LpsL6	-	349	7
GCF_014058625.1	618/90	1990	Ruminant	Bovine	United_Kingdom	CapA	LpsL6	-	349	7
GCF_014058645.1	619/90	1990	Ruminant	Bovine	United_Kingdom	CapA	LpsL6	-	349	7
GCF_014058665.1	671/90	1990	Ruminant	Bovine	United_Kingdom	CapA	LpsL6	-	349	7
GCF_014338445.1	Ban-PM4	2017	Bird	Chicken	Bangladesh	CapB	LpsL2	-	122	44
GCF_014338465.1	Ban-PM7	2017	Bird	Chicken	Bangladesh	CapB	LpsL2	-	122	44
GCF_014495805.1	PS3536-1p	2017	Ruminant	Bovine	Switzerland	CapA	LpsL3	-	79	1
GCF_015070975.1	PMF0(ysq001).l	2019	Rabbit	Rabbit	China	CapACapF	LpsL3	-	9	12
GCF_015354635.1	PMUVET4	2007	Ruminant	Alpaca	Peru	CapA	LpsL6	toxA	321	229
GCF_015354645.1	PMUVET5	2007	Ruminant	Alpaca	Peru	CapA	LpsL6	toxA	321	229
GCF_015354675.1	PMUVET3	2007	Ruminant	Alpaca	Peru	CapA	LpsL6	toxA	321	229
GCF_015354685.1	PMUVET2	2007	Ruminant	Alpaca	Peru	CapA	LpsL6	toxA	321	229
GCF_015354715.1	PMUVET1	2007	Ruminant	Alpaca	Peru	CapA	LpsL6	toxA	321	229
GCF_015546645.1	PMS_14	2016	Ruminant	Ovine	India	CapA	LpsL6	toxA	288	80
GCF_015546695.1	PMS_19	2016	Ruminant	Ovine	India	CapA	LpsL3	-	222	81
GCF_015546705.1	PMS_2	2015	Ruminant	Ovine	India	CapA	LpsL6	toxA	288	101
GCF_016313205.1	PM-1	2020	Ruminant	Bovine	China	CapB	LpsL2	-	122	44
GCF_016458265.1	PM1	2012	Ruminant	Bovine	Pakistan	CapB	LpsL2	-	122	44

GCF_016820295.1	B:2_'Soron'	2009	Pig	Porcine	India	CapA	LpsL1	-	472	-
GCF_016916875.1	PM2	2014	Ruminant	Bovine	Pakistan	CapB	LpsL2	-	122	44
GCF_017815735.1	Tibet-Pm1	2018	Ruminant	Bovine	China	CapB	LpsL2	-	122	44
GCF_018139065.1	HuN001	2021	Human	Human	China	CapA	LpsL1	-	471	204
GCF_018162435.1	MOR19	2019	Ruminant	Ovine	Morocco	CapA	LpsL3	-	79	1
GCF_019134675.1	PMWSG-4	2018	Bird	Duck	China	CapA	LpsL1	-	129	128
GCF_019754255.1	Pm3	2020	Ruminant	Bovine	China	CapA	LpsL3	-	79	1
GCF_019754275.1	Pm64	2020	Ruminant	Bovine	China	-	LpsL3	-	79	1
GCF_020971725.1	IMT47951	2019	Ruminant	Bovine	Germany	CapA	LpsL3	-	474	206
GCF_021441925.1	SD001	2019	-	-	China	CapA	LpsL6	-	27	10
GCF_021474145.1	SD11	2019	Rabbit	Rabbit	China	CapACapF	LpsL3	-	9	12
GCF_022179625.1	Pm1	2017	-	-	Japan	CapA	LpsL3	-	13	3
GCF_022213185.1	PmBUFF2016HRY	2016	Ruminant	Bovine	India	CapB	LpsL2	-	122	44
GCF_022213195.1	Pm52HVVI	2000	Ruminant	Bovine	India	CapB	LpsL2	-	122	44
GCF_022436465.1	cs	2019	Pig	Porcine	China	CapA	LpsL3LpsL6	-	475	238
GCF_022488925.1	Pm-CS-D	2019	Pig	Porcine	China	CapA	LpsL1	-	129	128
GCF_022559605.1	CUL-TANUVAS_2020	2020	Ruminant	Bovine	India	CapB	LpsL2	-	122	44
GCF_022575965.1	DC2020	2020	Bird	Duck	Bangladesh	CapB	LpsL2	-	122	44
GCF_022637295.1	GS2020-X2	2020	Wildlife	Marmota_himalayana	China	-	LpsL3	-	-	182
GCF_022869035.1	NIVEDIPm32	2019	Ruminant	Bovine	India	CapB	LpsL2	-	122	44
GCF_022869065.1	NIVEDIPm34	2019	Ruminant	Bovine	India	CapB	LpsL2	-	122	44
GCF_022869075.1	NIVEDIPm35	2019	Ruminant	Bovine	India	CapB	LpsL2	-	122	44
GCF_023555455.1	B621	2010	Rabbit	Rabbit	-	CapACapF	LpsL3	-	9	12
GCF_023555475.1	B859	2015	Rabbit	Rabbit	-	CapA	LpsL3	-	312	243
GCF_023555505.1	B852	2015	Rabbit	Rabbit	-	CapA	LpsL3	-	9	25
GCF_023555535.1	B639	2011	Rabbit	Rabbit	-	CapA	LpsL3	-	204	12
GCF_023555545.1	B635	2011	Rabbit	Rabbit	-	CapA	LpsL6	-	302	239
GCF_023555575.1	B617	2009	Rabbit	Rabbit	-	CapA	LpsL6	-	74	10
GCF_023555595.1	B615	2010	Rabbit	Rabbit	-	CapA	LpsL6	-	298	225
GCF_023555605.1	B607	2011	Rabbit	Rabbit	-	CapACapF	LpsL3	-	9	244
GCF_023555635.1	B609	2010	Rabbit	Rabbit	-	CapA	LpsL3	-	204	12
GCF_023555655.1	B555	2011	Rabbit	Rabbit	-	CapACapF	LpsL3	-	301	240
GCF_023555675.1	B522	2010	Rabbit	Rabbit	-	CapD	LpsL6	-	50	11
GCF_023555695.1	B288	2006	Rabbit	Rabbit	-	CapA	LpsL3	-	301	240
GCF_023555715.1	P1662	-	Bird	Turkey	-	CapA	-	-	53	223
GCF_023555735.1	P2100	-	Bird	Turkey	-	CapA	LpsL6	-	27	10
GCF_023555755.1	P1591	-	Human	Human	-	-	LpsL7	-	54	246
GCF_023555775.1	P2225	-	Ruminant	Bovine	-	CapA	LpsL1	-	452	-

GCF_023555795.1	P1997	-	Wildlife	Hering_gull	-	-	LpsL4	-	40	241
GCF_023555815.1	CAPM6431	2001	Rabbit	Rabbit	-	CapACapF	LpsL3	-	9	25
GCF_023555825.1	P2192	1972	Bird	Chicken	-	-	LpsL4	-	40	29
GCF_023555855.1	VP243	-	Ruminant	Bovine	-	CapE	LpsL2	-	162	245
GCF_023555875.1	P1581	-	Wildlife	Pine_siskin	-	-	LpsL7	-	54	246
GCF_023555895.1	VP584	-	Ruminant	Bovine	-	CapE	LpsL2	-	162	245
GCF_023555915.1	VP583	-	Ruminant	Bovine	-	CapE	LpsL2	-	292	242
GCF_023555925.1	VP582	-	Ruminant	Bovine	-	CapE	LpsL2	-	162	242
GCF_023555945.1	P1234	-	Ruminant	Bovine	-	CapE	LpsL2	-	292	242
GCF_023555995.1	M1404	1922	Ruminant	Bovine	-	CapB	LpsL2	-	122	64
GCF_023556015.1	39535	-	Bird	Chicken	-	CapA	LpsL1	-	37	33
GCF_023556035.1	P2095	-	Bird	Turkey	-	CapA	LpsL5	-	159	130
GCF_023556055.1	36655	-	Bird	Duck	-	CapA	LpsL3	-	128	222
GCF_023556075.1	P2093	1971	Rabbit	Rabbit	-	CapA	LpsL6	-	298	225
GCF_023556095.1	32022	-	Bird	Goose	-	CapA	LpsL3	-	2	199
GCF_023556105.1	P2687	1974	Ruminant	Bovine	-	CapA	LpsL6	-	-	7
GCF_023556135.1	P1573	-	Human	Human	-	CapA	LpsL6	-	74	10
GCF_023556155.1	P2723	-	Bird	Turkey	-	-	LpsL8	-	52	247
GCF_023556175.1	P3016	1975	Rabbit	Rabbit	-	CapA	LpsL6	-	298	225
GCF_023556185.1	31772	-	Bird	Duck	-	CapA	LpsL1	-	129	128
GCF_023556195.1	P2117	1971	Rabbit	Rabbit	-	CapA	LpsL6	-	298	225
GCF_023556225.1	P1971	1971	Ruminant	Bovine	-	CapA	LpsL3	-	80	3
GCF_023556255.1	P1702	-	Bird	Turkey	-	CapA	LpsL2	-	156	224
GCF_023556285.1	P1933	1970	Ruminant	Bovine	-	CapA	LpsL3	-	80	3
GCF_023556355.1	P2441	1973	Rabbit	Rabbit	-	CapA	LpsL3	-	13	3
GCF_023556415.1	B622	2010	Rabbit	Rabbit	-	CapD	LpsL6	-	296	248
GCF_023556435.1	P903	-	Pig	Porcine	-	CapD	LpsL6	-	50	11
GCF_023572865.1	10159	2022	Bird	Avian	USA	CapA	LpsL1	-	237	249
GCF_023572885.1	10185	2022	Bird	Avian	USA	CapA	LpsL3	-	235	234
GCF_023572905.1	11205	2022	Bird	Avian	USA	CapACapF	LpsL3	-	476	250
GCF_023572925.1	10957	2022	Bird	Avian	USA	CapA	LpsL3	-	477	251
GCF_023572945.1	11020	2022	Bird	Avian	USA	CapA	LpsL3	-	478	252
GCF_023572985.1	11245	2022	Bird	Avian	USA	CapA	LpsL3	-	235	234
GCF_023573005.1	P1702	2022	Bird	Avian	USA	CapA	LpsL2	-	156	224
GCF_023573025.1	32985	2022	Bird	Avian	USA	CapA	LpsL1	-	61	91
GCF_023573045.1	P2192	2022	Bird	Avian	USA	-	LpsL4	-	40	29
GCF_023573065.1	P2095	2022	Bird	Avian	USA	CapA	LpsL5	-	159	130
GCF_023573085.1	33011	2022	Bird	Avian	USA	CapA	LpsL1	-	61	91
GCF_023573105.1	P1581	2022	Bird	Avian	USA	-	LpsL7	-	54	246

GCF_023573125.1	P2723	2022	Bird	Avian	USA	CapA	LpsL8	-	479	253
GCF_023586965.1	38725	-	Bird	Chicken	-	-	LpsL6	-	480	254
GCF_023587085.1	21317	-	Bird	Chicken	-	CapA	LpsL3	-	481	257
GCF_023587205.1	35564	-	Bird	Chicken	-	CapA	LpsL3	-	481	257
GCF_023587305.1	31971	-	Bird	Chicken	-	CapA	-	-	482	255
GCF_023587465.1	41060	-	Bird	Chicken	-	CapACapF	LpsL5	-	159	256
GCF_023587585.1	36502	-	Bird	Chicken	-	CapACapF	LpsL3	-	9	12
GCF_023587605.1	21275	-	Bird	Turkey	-	CapD	LpsL6	-	483	205
GCF_023587625.1	28606	-	Bird	Goose	-	CapA	LpsL3	-	438	84
GCF_023587645.1	34030	-	Bird	Chicken	-	CapA	LpsL3	-	3	258
GCF_023587665.1	40540	-	Bird	Turkey	-	CapA	LpsL6	-	206	7
GCF_023587685.1	29135	-	Bird	Turkey	-	CapA	LpsL6	-	74	10
GCF_023587705.1	29792	-	Bird	Turkey	-	CapA	LpsL6	-	74	10
GCF_023587725.1	39639	-	Bird	Chicken	-	CapA	LpsL3	-	35	259
GCF_024055495.1	P2100	2022	Bird	Avian	USA	CapA	LpsL6	-	27	10
GCF_024055505.2	P2237	2022	Bird	Avian	USA	CapD	LpsL6	toxA	50	11
GCF_025398205.1	BAUFCTA	2019	Bird	Chicken	Bangladesh	CapA	LpsL1	-	201	260
GCF_026108305.1	NIVEDIPm36	2019	Pig	Porcine	India	CapA	LpsL6	-	74	10
GCF_026108315.1	NIVEDIPm17	2019	Pig	Porcine	India	CapD	LpsL6	-	50	11
GCF_026153335.1	P5041881	2020	Pig	Porcine	United_Kingdom	CapD	LpsL6	-	50	11
GCF_026153375.1	P504190	2020	Pig	Porcine	United_Kingdom	CapD	LpsL6	-	50	11
GCF_026315065.1	PF5	2021	Rabbit	Rabbit	China	CapACapF	LpsL3	-	430	12
GCF_026315085.1	PF6	2022	Rabbit	Rabbit	China	CapACapF	LpsL3	-	428	191
GCF_026315125.1	PF7	2021	Rabbit	Rabbit	China	CapACapF	LpsL3	-	430	12
GCF_026315145.1	PF9	2020	Rabbit	Rabbit	China	CapACapF	LpsL3	-	428	191
GCF_026315165.1	PF10	2021	Rabbit	Rabbit	China	CapACapF	LpsL3	-	428	191
GCF_026315225.1	PF11	2022	Rabbit	Rabbit	China	CapACapF	LpsL3	-	430	193
GCF_026409225.1	PF12	2021	Rabbit	Rabbit	China	CapACapF	LpsL3	-	428	191
GCF_026409245.1	PF14	2021	Rabbit	Rabbit	China	CapACapF	LpsL3	-	428	191
GCF_026409265.1	PF16	2021	Rabbit	Rabbit	China	CapACapF	LpsL3	-	428	191
GCF_026409285.1	PF19	2021	Rabbit	Rabbit	China	CapACapF	LpsL3	-	430	193
GCF_026409305.1	PF18	2021	Rabbit	Rabbit	China	CapACapF	LpsL3	-	428	191
GCF_026409325.1	PF1	2020	Rabbit	Rabbit	China	CapACapF	LpsL3	-	428	191
GCF_026409345.1	PF17	2021	Rabbit	Rabbit	China	CapACapF	LpsL3	-	430	193
GCF_026409365.1	PF15	2022	Rabbit	Rabbit	China	CapACapF	LpsL3	-	431	192
GCF_026626315.1	PM785D1B	2022	Ruminant	Bovine	USA	CapA	LpsL3	-	79	1
GCF_026636255.1	PF8	2021	Rabbit	Rabbit	China	CapACapF	LpsL3	-	430	12
GCF_026723845.1	PF13	2021	Rabbit	Rabbit	China	CapACapF	LpsL3	-	431	192
GCF_026738975.1	Alim_FC_1002	2021	Bird	Chicken	Bangladesh	CapB	LpsL2	-	122	44

GCF_026739035.1	Alim_FC_1000	2020	Bird	Chicken	Bangladesh	CapB	LpsL2	-	122	44
GCF_026739075.1	Alim_FC_1001	2020	Bird	Chicken	Bangladesh	CapB	LpsL2	-	122	44
GCF_026805485.1	RCAD0259	2015	Bird	Duck	China	CapA	LpsL1	-	129	128
GCF_027086695.1	SDHB	2019	Ruminant	Deer	India	CapB	LpsL2	-	122	194
GCF_027951085.1	NIVEDIPm20	2019	Ruminant	Ovine	India	CapA	-	toxA	288	80
GCF_027951095.1	NIVEDIPm3	2018	Ruminant	Ovine	India	CapA	-	toxA	288	80
GCF_027951105.1	NIVEDIPm9	2019	Ruminant	Ovine	India	CapA	LpsL6	toxA	288	80
GCF_027951115.1	NIVEDIPm1	2018	Ruminant	Ovine	India	CapA	-	toxA	288	80
GCF_027951165.1	NIVEDIPm19	2019	Ruminant	Ovine	India	CapA	LpsL6	toxA	288	80
GCF_027951305.1	NIVEDIPm31	2019	Ruminant	Ovine	India	CapA	LpsL6	toxA	288	80
GCF_027951325.1	NIVEDIPm10	2019	Ruminant	Ovine	India	CapA	LpsL6	toxA	288	80
GCF_027951355.1	NIVEDIPm22	2019	Ruminant	Ovine	India	CapA	LpsL6	toxA	288	80
GCF_028437575.1	PM2	-	Ruminant	Alpaca	Peru	CapA	LpsL6	toxA	321	229
GCF_028437595.1	PM1	-	Wildlife	Pelican	Peru	-	LpsL6	-	484	261
GCF_028826065.1	Alim_FC_1003	2020	Bird	Chicken	Bangladesh	CapB	LpsL2	-	122	44
GCF_029224005.1	LXSS001	2022	Rabbit	Rabbit	China	CapA	LpsL6	-	302	-
GCF_029324745.1	D-71	1985	-	-	Russia	CapA	-	-	129	128
GCF_029324765.1	1231	1986	Pig	Porcine	Russia	CapA	LpsL6	-	74	10
GCF_029324775.1	T-80	1980	Ruminant	Ovine	Russia	CapA	LpsL6	-	74	10
GCF_029324785.1	T-80-D	1993	Ruminant	Ovine	Russia	CapD	LpsL6	toxA	50	11
GCF_029543265.1	BD1769	2021	Bird	Chicken	Japan	-	LpsL4	-	273	29
GCF_029762475.1	PM-3	2020	-	-	China	CapA	LpsL1	-	129	128
GCF_029852795.1	Neville1	2013	Wildlife	Tasmanian_devil	Australia	-	LpsL1	-	542	341
GCF_029873295.1	19BRD-057	2019	Ruminant	Bovine	Australia	CapA	LpsL3	-	394	159
GCF_029906265.1	19BRD-032	2019	Ruminant	Bovine	Australia	CapA	LpsL3	-	394	159
GCF_029906285.1	18BRD-001	2018	Ruminant	Bovine	Australia	CapA	LpsL3	-	394	159
GCF_030059805.1	P030653/2	2022	Pig	Porcine	United_Kingdom	CapD	LpsL6	-	50	11
GCF_030122645.1	P030653/1	2022	Pig	Porcine	United_Kingdom	CapD	LpsL6	-	50	11
GCF_030846735.1	P1933	1970	Ruminant	Bovine	USA	CapA	LpsL3	-	80	3
GCF_031188105.1	PM140	-	-	-	China	CapD	LpsL6		50	11
GCF_031432935.1	sample-B	2022	Ruminant	Bovine	China	CapB	LpsL2		122	44
GCF_031463095.1	AKS2021-HT3	2021	Wildlife	Marmota_himalayana	China	-	LpsL3		-	183
GCF_031583005.1	AKS2022-HT5	2022	Wildlife	Marmota_himalayana	China	-	LpsL3		-	203
GCF_031587475.1	W22-979.1	2022	-	-	New_Zealand	CapB	LpsL3		64	342
GCF_031587935.1	W22-3735.1	2022	-	-	New_Zealand	CapB	LpsL3		62	343
GCF_031588995.1	W22-2894-7	2022	-	-	New_Zealand	CapA	LpsL3		455	219
GCF_031589515.1	W16-1607.1	2016	-	-	New_Zealand	CapACapF	LpsL3		179	344
GCF_032027925.1	17BRD-035	2017	Ruminant	Bovine	Australia	CapA	LpsL3	-	394	159
GCF_032259785.1	102426	2010	Pig	Porcine	Germany	CapD	LpsL6		50	11

GCF_032260685.1	80176	2008	Pig	Porcine	Germany	CapA	LpsL6		74	10
GCF_032335635.1	HUCA_PM_874	2019	Human	Human	Spain	CapA	LpsL1		130	131
GCF_032335655.1	HUCA_PM_084	2017	Human	Human	Spain	CapA	LpsL3		-	36
GCF_032598635.1	RCAD0726	2018	Bird	Duck	China	CapACapF	LpsL3		176	-
GCF_032598655.1	RCAD0730	2018	Bird	Duck	China	CapA	LpsL3		71	-
GCF_032669925.1	AKS2021-HT67	2021	Wildlife	Marmota_himalayana	China	-	-		-	184
GCF_032677625.1	GS2020-R1	2020	Rabbit	Rabbit	China	-	LpsL3		-	183
GCF_033041955.1	PVNRTVU1	2020	Ruminant	Bovine	India	CapB	LpsL2		122	44
GCF_033795605.1	94PV	2021	Ruminant	Bovine	Spain	CapA	LpsL3		13	3
GCF_033795645.1	870CM	2022	Ruminant	Bovine	Spain	CapA	LpsL3		80	4
GCF_033795665.1	790CV	2022	Ruminant	Bovine	Spain	CapA	LpsL3		13	3
GCF_033795675.1	756CM	2022	Ruminant	Bovine	Spain	CapA	LpsL3		13	3
GCF_033795705.1	725CV	2022	Ruminant	Bovine	Spain	CapA	LpsL3		79	1
GCF_033795715.1	720CV	2022	Ruminant	Bovine	Spain	CapA	LpsL3		79	1
GCF_033795745.1	494PV	2021	Ruminant	Bovine	Spain	CapA	LpsL3		13	3
GCF_033795765.1	483PV	2021	Ruminant	Bovine	Spain	CapA	LpsL3		13	3
GCF_033795785.1	432PM	2021	Ruminant	Bovine	Spain	CapA	LpsL3		13	3
GCF_033795805.1	286PV	2021	Ruminant	Bovine	Spain	CapA	LpsL3		13	3
GCF_033795815.1	282PV	2021	Ruminant	Bovine	Spain	CapA	LpsL3		79	-
GCF_033795845.1	189CV	2021	Ruminant	Bovine	Spain	CapA	LpsL3		79	1
GCF_033795865.1	104PV	2021	Ruminant	Bovine	Spain	CapA	LpsL3		80	-
GCF_033795885.1	111PV	2021	Ruminant	Bovine	Spain	CapA	LpsL3		13	3
GCF_034652515.1	Pm_SHZ01	2022	Ruminant	Ovine	China	CapA	LpsL3	-	132	65
GCF_035220555.1	Pm1621	2012	Dog/Cat	Feline	Australia	-	LpsL3	-	265	156
GCF_035221255.1	Pm1618	2012	Dog/Cat	Feline	Australia	CapA	LpsL1	-	30	32
GCF_035222775.1	Past33	2019	Human	Human	Australia	-	LpsL2	-	525	318
GCF_035223645.1	Past29	2019	Human	Human	Australia	CapA	-	-	482	255
GCF_035224565.1	Past9	2015	Human	Human	Australia	CapA	LpsL1	-	37	33
GCF_035225465.1	Past6	2008	Human	Human	Australia	-	LpsL3	-	-	-
GCF_035226565.1	Past3	2013	Human	Human	Australia	CapACapF	LpsL3	-	179	344
GCF_035520575.1	PI32	2014	Dog/Cat	Canine	Australia	-	LpsL3	-	7	147
GCF_035520655.1	PI31	2019	Dog/Cat	Canine	Australia	CapA	-	-	30	32
GCF_035520695.1	Pmc-B	2012	Dog/Cat	Feline	Australia	CapA	-	-	30	-
GCF_035520755.1	Pmc-A	2012	Dog/Cat	Feline	Australia	CapA	LpsL1	-	30	-
GCF_035520775.1	Pm1622	2012	Dog/Cat	Feline	Australia	CapA	-	-	30	32
GCF_035520795.1	Pmc-C	2012	Dog/Cat	Feline	Australia	CapA	LpsL1	-	30	-
GCF_035520835.1	Pm1620	2012	Dog/Cat	Feline	Australia	CapA	LpsL3	-	20	159
GCF_035520875.1	Pm1617	2012	Dog/Cat	Feline	Australia	-	LpsL3	-	265	156
GCF_035520895.1	Past34	2019	Human	Human	Australia	CapA	-	-	-	-

GCF_035520915.1	Pm1613	2012	Dog/Cat	Feline	Australia	-	LpsL3	-	20	159
GCF_035520935.1	Pm1476	2011	Dog/Cat	Feline	Australia	-	-	-	264	-
GCF_035520955.1	Past31	2019	Human	Human	Australia	CapA	-	-	527	324
GCF_035520975.1	Past30	2019	Human	Human	Australia	CapA	-	-	-	-
GCF_035520995.1	Past28	2019	Human	Human	Australia	CapA	LpsL3	-	359	87
GCF_035521015.1	Past26	2018	Human	Human	Australia	CapA	-	-	527	324
GCF_035521035.1	Past22	2017	Human	Human	Australia	CapF	-	-	66	30
GCF_035521055.1	Past23	2018	Human	Human	Australia	-	-	-	36	262
GCF_035521075.1	Past19	2017	Human	Human	Australia	CapA	-	-	66	30
GCF_035521095.1	Past18	2017	Human	Human	Australia	CapA	-	-	-	-
GCF_035521115.1	Past13	2017	Human	Human	Australia	CapA	LpsL3	-	451	-
GCF_035521135.1	Past7	2011	Human	Human	Australia	CapA	LpsL3	-	451	-
GCF_035521155.1	Past11	2017	Human	Human	Australia	CapA	-	-	171	38
GCF_035521175.1	Past10	2017	Human	Human	Australia	CapA	-	-	36	262
GCF_035521195.1	Past5	2015	Human	Human	Australia	CapA	LpsL3	-	444	208
GCF_035521215.1	Past4	2014	Human	Human	Australia	CapA	-	-	37	33
GCF_035521255.1	Past1	2009	Human	Human	Australia	CapA	-	-	37	33
GCF_035521535.1	Pm1612	2012	Dog/Cat	Feline	Australia	CapA	LpsL3	-	20	159
GCF_035521575.1	Pm1616	2012	Dog/Cat	Feline	Australia	-	LpsL3	-	265	156
GCF_035666055.1	PM-1	2023	-	-	China	CapA	LpsL3	-	79	1
GCF_035666075.1	PM-2	2023	-	-	China	CapD	LpsL3	-	125	178
GCF_036347715.1	FCf147	2023	Wildlife	Swan	China	-	LpsL2	-	-	-
GCF_036348715.1	B_OL	2021	Ruminant	Deer	Russia	CapB	LpsL2	-	122	64
GCF_036348735.1	Mol	2021	Ruminant	Bovine	Russia	CapACapF	LpsL3	-	9	12
GCF_036348755.1	B_Kr	2021	Ruminant	Deer	Russia	CapB	LpsL2	-	122	64
GCF_036349155.1	Past15	2017	Human	Human	Australia	CapA	LpsL1	-	58	167
GCF_036416355.1	LH06	2016	Rabbit	Rabbit	China	CapACapF	LpsL3	-	346	-
GCF_036688525.1	PM147	2022	-	-	-	CapA	LpsL1	-	129	-
GCF_036688545.1	PM115	2022	-	-	-	CapA	LpsL1	-	129	-
GCF_036688585.1	PM148	2022	-	-	-	CapA	LpsL1	-	129	-
GCF_036688605.1	PM114	2022	-	-	-	CapA	LpsL1	-	129	-
GCF_036688635.1	PM02	2022	-	-	-	CapA	LpsL1	-	129	-
GCF_036688655.1	PM03	2022	-	-	-	CapA	LpsL1	-	129	-
GCF_036864055.1	13313	2023	Human	Human	Tunisia	-	LpsL3	-	283	-
GCF_037021625.1	PM1463	2011	Pig	Porcine	Australia	CapA	LpsL1	-	11	157
GCF_037113485.1	212516	2021	Pig	Porcine	Germany	CapD	LpsL6	-	50	11
GCF_039134805.1	P154	2023	-	-	China	CapD	LpsL6	-	50	11
GCF_039514605.1	Pm6	2020	Rabbit	Rabbit	China	CapA	LpsL6	-	74	10
GCF_039519125.1	Pm3	2020	Rabbit	Rabbit	China	CapA	LpsL3	-	-	276

GCF_041442165.1	190055	2019	Pig	Porcine	-	CapD	LpsL6	-	50	11
GCF_041897555.1	W24_252	2023	Wildlife	Penguin	-	CapA	LpsL3	-	-	-
GCF_900115485.1	ATCC_51689	-	-	-	-	CapA	LpsL1	-	65	42
GCF_900187275.1	NCTC10322	1962	Pig	Porcine	-	CapA	LpsL3	-	13	3
GCF_900454565.1	NCTC11620	-	-	-	-	-	LpsL7	-	54	246
GCF_900454645.1	NCTC8489	-	-	-	-	CapA	LpsL1	-	11	299
GCF_900454845.1	NCTC11995	-	-	-	-	CapA	LpsL3	-	15	43
GCF_900478175.1	NCTC10382	1964	Human	Human	-	CapA	LpsL1	-	130	131
GCF_900636625.1	NCTC10204	1960	Ruminant	Bovine	United_Kingdom	CapA	LpsL1	-	65	42
GCF_900638315.1	NCTC11619	1983	Human	Human	-	-	-	-	-	345
GCF_900638665.1	NCTC10323	1963	Ruminant	Bovine	Myanmar	CapB	LpsL2	-	122	44
SRR10485145	PM1339	2009	Bird	Chicken	Australia	CapA	LpsL3	-	20	159
SRR10485146	PM1338	2009	Bird	Chicken	Australia	CapA	LpsL3	-	20	159
SRR10485147	PM1768	2009	Bird	Chicken	Australia	CapA	LpsL3	-	20	159
SRR10485148	PM1767	2009	Bird	Chicken	Australia	CapA	LpsL3	-	20	159
SRR10485149	PM1766	2009	Bird	Chicken	Australia	CapA	LpsL3	-	20	159
SRR10485150	PM1779	2013	Bird	Chicken	Australia	CapA	LpsL1	-	20	159
SRR10485151	PM1765	2013	Bird	Chicken	Australia	CapA	LpsL1	-	20	159
SRR10485152	PM1337	2009	Bird	Chicken	Australia	CapA	LpsL3	-	20	159
SRR10485153	PM1764	2013	Bird	Chicken	Australia	CapA	-	-	20	159
SRR10485154	PM1763	2013	Bird	Chicken	Australia	CapA	LpsL1	-	20	159
SRR10485155	PM1762	2013	Bird	Chicken	Australia	CapA	LpsL1	-	20	159
SRR10485156	PM1761	2013	Bird	Chicken	Australia	CapA	LpsL1	-	20	159
SRR10485157	PM1760	2013	Bird	Chicken	Australia	CapA	LpsL1	-	20	159
SRR10485158	PM1759	2013	Bird	Chicken	Australia	CapA	LpsL1	-	20	159
SRR10485159	PM1758	2013	Bird	Chicken	Australia	CapA	LpsL1	-	20	159
SRR10485160	PM1757	2013	Bird	Chicken	Australia	CapA	LpsL1	-	20	159
SRR10485161	PM1756	2013	Bird	Chicken	Australia	CapA	LpsL1	-	20	159
SRR10485162	PM1754	2013	Bird	Chicken	Australia	CapA	LpsL1	-	20	159
SRR10485163	PM1336	2009	Bird	Chicken	Australia	CapA	LpsL3	-	20	159
SRR10485164	PM1711	2013	Bird	Chicken	Australia	CapA	LpsL1	-	20	159
SRR10485165	PM1710	2013	Bird	Chicken	Australia	CapA	LpsL1	-	20	159
SRR10485166	PM1709	2013	Bird	Chicken	Australia	CapA	LpsL1	-	20	159
SRR10485167	PM1708	2013	Bird	Chicken	Australia	CapA	LpsL1	-	20	159
SRR10485168	PM1707	2013	Bird	Chicken	Australia	CapA	LpsL1	-	20	159
SRR10485169	PM1651	2012	Bird	Chicken	Australia	CapA	LpsL1	-	20	159
SRR10485170	PM1650	2012	Bird	Chicken	Australia	CapA	LpsL1	-	20	159
SRR10485171	PM1649	2012	Bird	Chicken	Australia	CapA	LpsL1	-	20	159
SRR10485172	PM1647	2012	Bird	Chicken	Australia	CapA	LpsL1	-	20	159

SRR10485173	PM1646	2012	Bird	Chicken	Australia	CapA	LpsL1	-	20	159
SRR10485174	PM1335	2009	Bird	Chicken	Australia	CapA	LpsL3	-	20	159
SRR10485175	PM1645	2012	Bird	Chicken	Australia	CapA	LpsL1	-	20	159
SRR10485176	PM1644	2012	Bird	Chicken	Australia	CapA	LpsL1	-	20	159
SRR10485177	PM1643	2012	Bird	Chicken	Australia	CapA	LpsL1	-	20	159
SRR10485178	PM1641	2012	Bird	Chicken	Australia	CapA	LpsL1	-	20	159
SRR10485179	PM1639	2012	Bird	Chicken	Australia	CapA	LpsL1	-	20	159
SRR10485180	PM1638	2012	Bird	Chicken	Australia	CapA	LpsL1	-	20	159
SRR10485181	PM1637	2012	Bird	Chicken	Australia	CapA	LpsL1	-	20	159
SRR10485182	PM1636	2012	Bird	Chicken	Australia	CapA	LpsL1	-	20	159
SRR10485183	PM1635	2012	Bird	Chicken	Australia	CapA	LpsL1	-	20	159
SRR10485184	PM1634	2012	Bird	Chicken	Australia	CapA	LpsL1	-	20	159
SRR10485185	PM1334	2009	Bird	Chicken	Australia	CapA	LpsL3	-	20	159
SRR10485186	PM1615	2012	Bird	Chicken	Australia	CapA	LpsL1	-	20	159
SRR10485187	PM1614	2012	Bird	Chicken	Australia	CapA	LpsL1	-	20	159
SRR10485188	PM1430	2010	Bird	Chicken	Australia	CapA	LpsL3	-	20	159
SRR10485189	PM1429	2010	Bird	Chicken	Australia	CapA	LpsL3	-	20	159
SRR10485190	PM1428	2010	Bird	Chicken	Australia	CapA	LpsL3	-	20	159
SRR10485191	PM1427	2010	Bird	Chicken	Australia	CapA	LpsL3	-	20	159
SRR10485192	PM1426	2010	Bird	Chicken	Australia	CapA	LpsL3	-	20	159
SRR10485193	PM1425	2010	Bird	Chicken	Australia	CapA	LpsL3	-	20	159
SRR10485194	PM1424	2010	Bird	Chicken	Australia	CapA	LpsL3	-	20	159
SRR10485195	PM1423	2010	Bird	Chicken	Australia	CapA	LpsL3	-	20	159
SRR10485196	PM1333	2009	Bird	Chicken	Australia	CapA	LpsL3	-	20	159
SRR10485197	PM1422	2010	Bird	Chicken	Australia	CapA	LpsL3	-	20	159
SRR10485198	PM1358	2009	Bird	Chicken	Australia	CapA	LpsL3	-	-	-
SRR10485199	PM1357	2009	Bird	Chicken	Australia	CapA	LpsL3	-	20	159
SRR10485200	PM1356	2009	Bird	Chicken	Australia	CapA	LpsL3	-	20	159
SRR10485201	PM1355	2009	Bird	Chicken	Australia	CapA	LpsL3	-	20	159
SRR10485202	PM1354	2009	Bird	Chicken	Australia	CapA	LpsL3	-	20	159
SRR10485203	PM1353	2009	Bird	Chicken	Australia	CapA	LpsL3	-	20	159
SRR10485204	PM1352	2009	Bird	Chicken	Australia	CapA	LpsL3	-	20	159
SRR10485205	PM1351	2009	Bird	Chicken	Australia	CapA	LpsL3	-	20	159
SRR10485206	PM1350	2009	Bird	Chicken	Australia	CapA	LpsL3	-	20	159
SRR10485207	PM1332	2009	Bird	Chicken	Australia	CapA	LpsL3	-	20	159
SRR10485208	PM1349	2009	Bird	Chicken	Australia	CapA	LpsL3	-	20	159
SRR10485209	PM1348	2009	Bird	Chicken	Australia	CapA	LpsL3	-	20	159
SRR10485210	PM1347	2009	Bird	Chicken	Australia	CapA	LpsL3	-	20	159
SRR10485211	PM1346	2009	Bird	Chicken	Australia	CapA	LpsL3	-	20	159

SRR10485212	PM1345	2009	Bird	Chicken	Australia	CapA	LpsL3	-	20	159
SRR10485213	PM1344	2009	Bird	Chicken	Australia	CapA	LpsL3	-	20	159
SRR10485214	PM1343	2009	Bird	Chicken	Australia	CapA	LpsL3	-	20	159
SRR10485215	PM1342	2009	Bird	Chicken	Australia	CapA	LpsL3	-	20	159
SRR10485216	PM1341	2009	Bird	Chicken	Australia	CapA	LpsL3	-	20	159
SRR10485217	PM1340	2009	Bird	Chicken	Australia	CapA	LpsL3	-	20	159
SRR10485218	PM1331	2009	Bird	Chicken	Australia	CapA	LpsL3	-	20	159
SRR10485219	PM1330	2009	Bird	Chicken	Australia	CapA	LpsL3	-	20	159
SRR12130698	PM213	1994	Bird	Chicken	Australia	CapA	LpsL3	-	20	159
SRR12130699	PM212	1994	Bird	Chicken	Australia	CapA	LpsL3	-	20	159
SRR12130700	PM2587	2019	Bird	Chicken	Australia	CapA	LpsL3	-	9	151
SRR12130701	PM2586	2019	Bird	Chicken	Australia	CapA	LpsL3	-	9	151
SRR12130702	PM2585	2019	Bird	Chicken	Australia	CapA	LpsL3	-	9	151
SRR12130703	PM211	1994	Bird	Chicken	Australia	CapA	LpsL3	-	20	159
SRR12130704	PM2470	2018	Bird	Chicken	Australia	CapA	LpsL3	-	9	151
SRR12130705	PM2445	2018	Bird	Chicken	Australia	CapA	LpsL3	-	9	151
SRR12130706	PM2401	2018	Bird	Chicken	Australia	CapA	LpsL3	-	9	151
SRR12130707	PM2398	2018	Bird	Chicken	Australia	CapA	LpsL3	-	9	151
SRR12130708	PM2391	2018	Bird	Chicken	Australia	CapA	LpsL3	-	9	151
SRR12130709	PM2390	2018	Bird	Chicken	Australia	CapA	LpsL3	-	9	151
SRR12130710	PM2389	2018	Bird	Chicken	Australia	CapA	LpsL3	-	9	151
SRR12130711	PM2388	2018	Bird	Chicken	Australia	CapA	LpsL3	-	9	151
SRR12130712	PM2387	2018	Bird	Chicken	Australia	CapA	LpsL3	-	9	151
SRR12130713	PM2386	2018	Bird	Chicken	Australia	CapA	LpsL3	-	9	151
SRR12130714	PM210	1994	Bird	Chicken	Australia	CapA	LpsL3	-	20	159
SRR12130715	PM2385	2018	Bird	Chicken	Australia	CapA	LpsL3	-	9	151
SRR12130716	PM2384	2018	Bird	Chicken	Australia	-	LpsL3	-	9	151
SRR12130717	PM2383	2018	Bird	Chicken	Australia	CapA	LpsL3	-	9	151
SRR12130718	PM2369	2017	Bird	Chicken	Australia	CapA	LpsL3	-	9	151
SRR12130719	PM2356	2017	Bird	Chicken	Australia	CapA	LpsL3	-	20	159
SRR12130720	PM2305	2016	Bird	Chicken	Australia	CapA	LpsL3	-	9	151
SRR12130721	PM2290	2016	Bird	Chicken	Australia	CapA	LpsL3	-	7	147
SRR12130722	PM2269	2016	Bird	Chicken	Australia	CapA	LpsL3	-	9	151
SRR12130723	PM2248	2016	Bird	Chicken	Australia	CapA	LpsL3	-	20	159
SRR12130724	PM1855	2014	Bird	Chicken	Australia	CapA	LpsL3	-	8	124
SRR12130725	PM209	1994	Bird	Chicken	Australia	CapA	LpsL3	-	20	159
SRR12130726	PM1853	2014	Bird	Chicken	Australia	CapA	LpsL3	-	8	124
SRR12130727	PM1852	2014	Bird	Chicken	Australia	CapA	LpsL3	-	8	124
SRR12130728	PM1851	2014	Bird	Chicken	Australia	CapA	LpsL3	-	8	124

SRR12130729	PM1850	2014	Bird	Chicken	Australia	CapA	LpsL3	-	8	124
SRR12130730	PM1849	2014	Bird	Chicken	Australia	CapA	LpsL3	-	8	124
SRR12130731	PM1848	2014	Bird	Chicken	Australia	CapA	LpsL3	-	8	124
SRR12130732	PM1847	2014	Bird	Chicken	Australia	CapA	LpsL3	-	8	124
SRR12130733	PM1846	2014	Bird	Chicken	Australia	CapA	LpsL3	-	8	124
SRR12130734	PM1845	2014	Bird	Chicken	Australia	CapA	LpsL3	-	8	124
SRR12130735	PM1582	2012	Bird	Chicken	Australia	CapA	LpsL3	-	451	209
SRR12130736	PM208	1994	Bird	Chicken	Australia	CapA	LpsL3	-	20	159
SRR12130737	PM1541	2011	Bird	Chicken	Australia	CapA	LpsL3	-	451	209
SRR12130738	PM1447	2010	Bird	Chicken	Australia	CapA	LpsL3	-	451	209
SRR12130739	PM844	2001	Bird	Chicken	Australia	CapA	LpsL3	-	8	124
SRR12130740	PM842	2001	Bird	Chicken	Australia	CapA	LpsL3	-	20	159
SRR12130741	PM2240	2016	Bird	Chicken	Australia	CapA	LpsL3	-	9	151
SRR12130742	PM2239	2016	Bird	Chicken	Australia	CapA	LpsL3	-	9	151
SRR12130743	PM2238	2016	Bird	Chicken	Australia	CapA	LpsL3	-	9	151
SRR12130744	PM2237	2016	Bird	Chicken	Australia	CapA	LpsL3	-	9	151
SRR12130745	PM2236	2016	Bird	Chicken	Australia	CapA	LpsL3	-	9	151
SRR12130746	PM2235	2016	Bird	Chicken	Australia	CapA	LpsL3	-	9	151
SRR12130747	PM207	1994	Bird	Chicken	Australia	CapA	LpsL3	-	20	159
SRR12130748	PM2234	2016	Bird	Chicken	Australia	CapA	LpsL3	-	9	151
SRR12130749	PM2233	2016	Bird	Chicken	Australia	CapA	LpsL3	-	9	151
SRR12130750	PM2232	2016	Bird	Chicken	Australia	CapA	LpsL3	-	9	151
SRR12130751	PM2142	2015	Bird	Chicken	Australia	CapA	LpsL3	-	9	151
SRR12130752	PM1949	2014	Bird	Chicken	Australia	CapA	LpsL3	-	9	151
SRR12130753	PM1948	2014	Bird	Chicken	Australia	CapA	LpsL3	-	9	151
SRR12130754	PM1947	2014	Bird	Chicken	Australia	CapA	LpsL3	-	9	151
SRR12130755	PM1778	2013	Bird	Chicken	Australia	CapA	LpsL3	-	9	151
SRR12130756	PM1777	2013	Bird	Chicken	Australia	CapA	LpsL3	-	9	151
SRR12130757	PM1776	2013	Bird	Chicken	Australia	CapA	LpsL3	-	9	151
SRR12130758	PM206	1994	Bird	Chicken	Australia	CapA	LpsL3	-	20	159
SRR12130759	PM1775	2013	Bird	Chicken	Australia	CapA	LpsL3	-	9	151
SRR12130760	PM1542	2011	Bird	Chicken	Australia	CapA	LpsL3	-	9	151
SRR12130761	PM1533	2011	Bird	Chicken	Australia	CapA	LpsL3	-	9	151
SRR12130762	PM984	2002	Bird	Chicken	Australia	CapA	LpsL3	-	20	159
SRR12130763	PM983	2002	Bird	Chicken	Australia	CapA	LpsL3	-	20	159
SRR12130764	PM982	2002	Bird	Chicken	Australia	CapA	LpsL3	-	20	159
SRR12130765	PM981	2002	Bird	Chicken	Australia	CapA	LpsL3	-	20	159
SRR12130766	PM980	2002	Bird	Chicken	Australia	CapA	LpsL3	-	20	159
SRR12130767	PM979	2002	Bird	Chicken	Australia	CapA	LpsL3	-	20	159

SRR12130768	PM214	1994	Bird	Chicken	Australia	CapA	LpsL3	-	20	159
SRR12130769	PM205	1994	Bird	Chicken	Australia	CapA	LpsL3	-	20	159
SRR12130770	PM204	1994	Bird	Chicken	Australia	CapA	LpsL3	-	20	159
SRR9051182	PM2192	2016	Wildlife	Gliding_possum	Australia	-	LpsL4	-	10	218
SRR9051183	PM2190	2016	Wildlife	Gliding_possum	Australia	-	-	-	10	218
SRR9051184	PM2191	2016	Wildlife	Gliding_possum	Australia	-	LpsL4	-	10	218
SRR9051185	PM2183	2015	Wildlife	Gliding_possum	Australia	-	LpsL4	-	10	217
SRR9051186	PM2194	2016	Wildlife	Rat-kangaroo	Australia	-	-	-	10	220
SRR9051187	PM2346	2017	Wildlife	Woylie	Australia	CapF	LpsL3	-	24	161
SRR9051188	PM2182	2015	Wildlife	Gliding_possum	Australia	-	-	-	10	217
SRR9051189	PM2193	2016	Wildlife	Gliding_possum	Australia	-	-	-	10	218
SRR9051190	PM2188	2016	Wildlife	Gliding_possum	Australia	-	LpsL4	-	10	218
SRR9051191	PM2189	2016	Wildlife	Gliding_possum	Australia	-	LpsL4	-	10	218
SRR9051192	PM2186	2015	Wildlife	Woylie	Australia	CapA	-	-	306	-
SRR9051193	PM2187	2016	Wildlife	Gliding_possum	Australia	-	LpsL4	-	10	217
SRR9051194	PM2184	2015	Wildlife	Gliding_possum	Australia	-	LpsL4	-	10	218
SRR9051195	PM2185	2015	Wildlife	Gliding_possum	Australia	-	LpsL4	-	10	218
GCA_035619375.1	CM2009-0393-0	2009	Bird	Chicken	Australia	CapA	LpsL1	-	20	159
GCA_035619395.1	CM2013-0203-0	2013	Wildlife	Chestnut_Teal	Australia	CapA	LpsL3	-	20	159
GCA_035619355.1	CM2013-0425-0	2013	Wildlife	Swan	Australia	CapA	LpsL3	-	20	159
GCA_035619335.1	CM2014-0810-0	2014	Bird	Chicken	Australia	CapA	LpsL1	-	20	159
GCA_035619315.1	CM2015-0868-0	2015	Bird	Chicken	Australia	CapA	LpsL3	-	20	159
GCA_035619295.1	CM2016-1019-2	2016	Bird	Chicken	Australia	CapA	LpsL1	-	20	159
GCA_035619255.1	CM2016-1054-3	2016	Bird	Chicken	Australia	CapA	LpsL1	-	20	159
GCA_035619275.1	CM2016-1054-4	2016	Bird	Chicken	Australia	CapA	LpsL1	-	20	159
GCA_035619215.1	CM2016-1086-0	2016	Bird	Chicken	Australia	CapA	LpsL1	-	20	159
GCA_035619235.1	CM2016-1086-1	2016	Bird	Chicken	Australia	CapA	LpsL1	-	20	159
GCA_035619195.1	CM2017-0502-0	2017	Bird	Chicken	Australia	CapA	LpsL1	-	20	159
GCA_035619175.1	CM2017-0502-1	2017	Bird	Chicken	Australia	CapA	LpsL1	-	20	159
GCA_035619135.1	CM2017-0740-0	2017	Bird	Chicken	Australia	CapA	LpsL3	-	20	159
GCA_035619155.1	CM2017-0740-1	2017	Bird	Chicken	Australia	CapA	LpsL3	-	20	159
GCA_035619115.1	CM2017-0740-2	2017	Bird	Chicken	Australia	CapA	LpsL3	-	20	159
GCA_035619095.1	CM2017-0740-3	2017	Bird	Chicken	Australia	CapA	LpsL1	-	20	159
GCA_035619075.1	CM2017-0740-4	2017	Bird	Chicken	Australia	CapA	LpsL1	-	20	159
GCA_035619055.1	CM2017-1139-0	2017	Bird	Chicken	Australia	CapA	LpsL1	-	20	159
GCA_035618995.1	CM2017-1139-1	2017	Bird	Chicken	Australia	CapA	LpsL1	-	20	159
GCA_035619015.1	CM2021-0180-0	2021	Bird	Turkey	Australia	CapA	LpsL1	-	20	159
GCA_035619035.1	CM2021-0180-1	2021	Bird	Turkey	Australia	CapA	LpsL1	-	20	159
GCA_035618975.1	CM2021-0180-2	2021	Bird	Turkey	Australia	CapA	LpsL1	-	20	159

GCA_035618955.1	CM2022-0138-0	2022	Bird	Turkey	Australia	CapA	LpsL3	-	20	159
GCA_035618895.1	CM2022-0189-1	2022	Bird	Chicken	Australia	CapA	LpsL3	-	20	159
GCA_035618915.1	CM2022-0189-2	2022	Bird	Chicken	Australia	CapA	LpsL3	-	20	159

S3 Table. List of the 94 RefSeq genomes from the *Pasteurellaceae* family used to confirm taxonomic identification.

Accession	Species	Strain	Level of completion
GCF_001647695.1	[*Haemophilus*] *ducreyi*	VAN2	Complete
GCF_000007745.1	[*Mannheimia*] *succiniciproducens*	MBEL55E	Complete
GCF_000374285.1	*Actinobacillus capsulatus*	DSM 19761	Scaffold
GCF_900638385.1	*Actinobacillus delphinicola*	NCTC12871	Complete
GCF_000801145.1	*Actinobacillus equuli* subsp. *equuli*	19392	Complete
GCF_901764975.1	*Actinobacillus indolicus*	46K2C	Contig
GCF_900444945.1	*Actinobacillus lignieresii*	NCTC4189	Contig
GCF_000175195.1	*Actinobacillus minor*	NM305	Contig
GCF_900638445.1	*Actinobacillus pleuropneumoniae*	NCTC10976	Complete
GCF_901764995.1	*Actinobacillus porcinus*	NM319	Scaffold
GCF_003101015.1	*Actinobacillus porcitonsillarum*	9953L55	Complete
GCF_900460625.1	*Actinobacillus seminis*	NCTC10851	Contig
GCF_002591855.1	*Actinobacillus succinogenes*	GXAS137	Scaffold
GCF_000739435.1	*Actinobacillus suis*	ATCC 33415	Complete
GCF_900445015.1	*Actinobacillus ureae*	NCTC10220	Contig
GCF_901687125.1	*Actinobacillus vicugnae*	W16181	Contig
GCF_023518055.1	*Aggregatibacter actinomycetemcomitans*	4S	Complete
GCF_900636915.1	*Aggregatibacter aphrophilus*	NCTC 5906	Complete
GCF_003130255.1	*Aggregatibacter kilianii*	PN_528	Contig
GCF_900476035.1	*Aggregatibacter segnis*	NCTC 10977	Complete
GCF_900454535.1	*Avibacterium avium*	NCTC 11297	Contig
GCF_002921145.1	*Avibacterium endocarditidis*	20186H4H1	Scaffold
GCF_004362535.1	*Avibacterium gallinarum*	DSM 17481	Scaffold
GCF_011765605.1	*Avibacterium paragallinarum*	ESV-135	Complete
GCF_900635775.1	*Avibacterium volantium*	NCTC3438	Complete
GCF_011455875.1	*Basfia succiniciproducens*	JF4016	Complete
GCF_000521725.1	*Bibersteinia trehalosi*	USDA-ARS-USMARC-188	Complete
GCF_013377195.1	*Bisgaardia hudsonensis*	M327/99/2	Complete
GCF_900450725.1	*Canicola haemoglobinophilus*	NCTC1659	Contig
GCF_002795405.1	*Caviibacterium pharyngocola*	7.3	Contig
GCF_000772535.1	*Chelonobacter oris*	1662	Contig
GCF_002795425.1	*Conservatibacter flavescens*	7.4	Contig
GCF_004340985.1	*Cricetibacter osteomyelitidis*	DSM 28404	Contig
GCF_011455495.1	*Frederiksenia canicola*	HPA 21	Complete
GCF_000772265.1	*Gallibacterium anatis*	F149	Contig
GCF_001678495.1	*Gallibacterium salpingitidis*	F150	Contig

GCF_003260095.1	*Glaesserella australis*	HS4635	Scaffold
GCF_017352235.1	*Glaesserella parasuis*	YHP1818	Complete
GCF_900475885.1	*Haemophilus aegyptius*	NCTC8502	Complete
GCF_900477945.1	*Haemophilus haemolyticus*	NCTC10839	Complete
GCF_000931575.1	*Haemophilus influenzae*	477	Complete
GCF_002015115.1	*Haemophilus paracuniculus*	CCUG 43573	Scaffold
GCF_016889385.1	*Haemophilus parahaemolyticus*	FDAARGOS_1199	Complete
GCF_000191405.1	*Haemophilus parainfluenzae*	ATCC 33392	Scaffold
GCF_900451065.1	*Haemophilus paraphrohaemolyticus*	NCTC10671	Contig
GCF_900186995.1	*Haemophilus pittmaniae*	NCTC13334	Complete
GCF_008605885.1	*Haemophilus seminalis*	SZY H2	Scaffold
GCF_000287615.1	*Haemophilus sputorum*	HK 2154	Contig
GCF_000019405.1	*Histophilus somni*	2336	Complete
GCF_004339625.1	*Lonepinella koalarum*	DSM 10053	Scaffold
GCF_014541205.1	*Mannheimia bovis*	ZY190616	Complete
GCF_011455695.1	*Mannheimia granulomatis*	B 234/94	Complete
GCF_002285575.1	*Mannheimia haemolytica*	USDA-ARS-USMARC-191	Complete
GCF_000940515.1	*Mannheimia massilioguelmaensis*	MG13T	Contig
GCF_009828705.1	*Mannheimia ovis*	ZY170218	Complete
GCF_013378015.1	*Mannheimia pernigra*	16CN0041	Complete
GCF_000521695.1	*Mannheimia varigena*	USDA-ARS-USMARC-1312	Complete
GCF_003265225.1	*Mergibacter septicus*	27517-4-I1	Complete
GCF_004363295.1	*Mesocricetibacter intestinalis*	DSM 28403	Scaffold
GCF_004569585.1	*Muribacter muris*	WT12	Contig
GCF_000827595.2	*Necropsobacter massiliensis*	FF6	Scaffold
GCF_004345745.1	*Nicoletella semolina*	DSM 16380	Scaffold
GCF_009684715.1	*Otariodibacter oris*	Baika1	Complete
GCF_018343795.1	*Pasteurella atlantica*	NVI-9100	Complete
GCF_000262245.1	*Pasteurella bettyae*	CCUG 2042	Contig
GCF_024622045.1	*Pasteurella caecimuris*	DSM 28627	Contig
GCF_020810675.1	*Pasteurella canis*	HL_NV12211	Complete
GCF_900186835.1	*Pasteurella dagmatis*	NCTC11617	Complete
GCF_003096995.1	*Pasteurella langaaensis*	DSM 22999	Contig
GCF_002073255.2	*Pasteurella multocida*	FDAARGOS_218	Complete
GCF_002850605.1	*Pasteurella oralis*	WCHPO000540	Contig
GCF_013377295.1	*Pasteurella skyensis*	95A1	Complete
GCF_900454705.1	*Pasteurella testudinis*	NCTC12150	Contig
GCF_900454895.1	*Phocoenobacter uteri*	NCTC12872	Contig
GCF_003585965.1	*Psittacicella gerlachiana*	EEAB3T1	Contig

GCF_003585935.1	*Psittacicella hinzii*	111	Contig
GCF_003585925.1	*Psittacicella melopsittaci*	B96_4	Contig
GCF_015356115.1	*Rodentibacter haemolyticus*	DSM 111151	Complete Genome
GCF_002000125.1	*Rodentibacter heidelbergensis*	Ac69	Contig
GCF_010587025.1	*Rodentibacter heylii*	G1	Complete Genome
GCF_001998825.1	*Rodentibacter mrazii*	Ppn418	Contig
GCF_001999305.1	*Rodentibacter myodis*	Ac151	Contig
GCF_000730685.1	*Rodentibacter pneumotropicus*	ATCC 35149	Contig
GCF_001998965.1	*Rodentibacter rarus*	CCUG 17206	Contig
GCF_002000485.1	*Rodentibacter ratti*	F75	Contig
GCF_002000425.1	*Rodentibacter trehalosifermentans*	H1983213011	Contig
GCF_014885015.1	*Spirabiliibacterium falconis*	NCTC 11878	Contig
GCF_014884965.1	*Spirabiliibacterium mucosae*	20609_3	Contig
GCF_014884995.1	*Spirabiliibacterium pneumoniae*	HPA106	Contig
GCF_004342725.1	*Testudinibacter aquarius*	DSM 28140	Scaffold
GCF_009761375.1	*Ursidibacter arcticus*	Bamse1	Contig
GCF_009761395.1	*Ursidibacter maritimus*	Pb43106	Contig
GCF_013377275.1	*Vespertiliibacter pulmonis*	CCUG 64585	Chromosome
GCF_004339025.1	*Volucribacter psittacicida*	DSM 15534	Scaffold

S4 Table. Country and host distribution of whole genome sequences of *P. multocida* analysed using PopPUNK.

Data Source	Country	Domestic Bird	Ruminant	Pig	Rabbit	Dog or Cat	Human	Wildlife	Not specified	Horse	Environment	# genomic sequences by country & data source
	Australia	23	4	1		14	22	3				67
	Bangladesh	8	1									9
	Canada		1									1
	China	18	12	58	26		1	5	13			133
	France				17							17
	Germany		1	3								4
	Greece					1	1					2
	India		25	3								28
	Iran		1									1
	Japan	1							1			2
	Kazakhstan		1							2		3
	Malaysia						1		1			2
	Morocco		1									1
	Myanmar		1									1
GenBank	Netherlands						1					1
	New Zealand								4			4
	Not specified	30	13	5	18		4	3	18			91
	Pakistan		11						1			12
	Peru		8					1				9
	Russia		5	1					1			7
	South Korea					1						1
	Spain		14				2					16
	Sri Lanka		1									1
	Switzerland		1									1
	Thailand		3									3
	United Kingdom		14	4								18
	USA	22	57			1	4				2	86
	Vietnam	1										1
	Tunisia						1					1
SRA	Australia	148						14				162
This study	Australia	18	10	1	5	20		4			1	59

# genomic sequences by host		270	185	76	66	37	37	30	39	2	3	744
