# Supplementary material for: Comparative genome analysis of Pasteurella multocida from Australian domestic animals suggests broad patterns of transmissions across multiple hosts and origins
Source: PLoS One. 2025 Aug 6;20(8):e0329807. doi: 10.1371/journal.pone.0329807 (PMC12327604; doi:10.1371/journal.pone.0329807)
Supplement: S3 Table — (PDF) [file pone.0329807.s003.pdf]

S3 Table. Genome sequencing statistics for *P. multocida* isolates from this study.

Isolate	Assembly format	Assembly type	num_seqs_R1	sum_len_R1	N50_R1	Q20(%)_R1	Q30(%)_R1	num_seqs_R2	sum_len_R2	N50_R2	Q20(%)_R2	Q30(%)_R2	Assembly_contigs	Assembly_sum_len	Assembly_min_len	Assembly_avg_len	Assembly_max_len	Assembly_N50	Assembly_Depth
CM2006-0650-0	FASTA	DNA	2353881	292768358	125	96.33	92.02	2353881	292171541	125	95.26	90.73	46	2274574	103	49447.3	513894	484200	257
CM2007-0542-0	FASTA	DNA	2398352	298345118	125	96.37	92.07	2398352	297813511	125	95.48	91.1	27	2233878	124	82736.2	528153	448431	267
CM2009-0453-0	FASTA	DNA	2456853	305560126	125	96.67	92.8	2456853	304659150	125	94.04	88.67	40	2328521	103	58213	647808	389379	262
CM2009-0556-0	FASTA	DNA	2567415	385103795	151	97.98	93.8	2567415	384646207	151	93.5	84.92	48	2287750	115	47661.5	764339	505892	336
CM2009-0682-0	FASTA	DNA	1928678	289570940	151	97.84	93.24	1928678	289210161	151	92.98	84.09	43	2282528	118	53082	606411	449047	254
CM2009-0827-0	FASTA	DNA	2860360	355674318	125	96.27	91.83	2860360	354822856	125	94.53	89.41	26	2321832	113	89301.2	459429	383723	306
CM2010-0113-1	FASTA	DNA	2229645	277305678	125	96.19	91.74	2229645	276340719	125	93.15	87.12	28	2272715	118	81168.4	480661	436457	244
CM2010-0286-0	FASTA	DNA	2328457	289639367	125	96.49	92.39	2328457	289039769	125	94.99	90.26	31	2301617	121	74245.7	610290	434424	251
CM2010-0516-0	FASTA	DNA	2367766	294483469	125	96.24	91.9	2367766	293864107	125	95.15	90.56	52	2243979	110	43153.4	605229	535255	262
CM2010-0545-2	FASTA	DNA	2670011	400988200	151	97.34	91.79	2670011	400587122	151	93.13	84.2	24	2285167	128	95215.3	608927	302115	351
CM2011-0151-1	FASTA	DNA	2096201	314373132	151	98.01	93.58	2096201	313998223	151	93.06	84.29	23	2274908	108	98909	533536	294344	276
CM2011-0458-1	FASTA	DNA	2321807	348523573	151	97.99	93.69	2321807	348089999	151	92.88	83.89	27	2428371	107	89939.7	634129	430329	287
CM2011-0584-0	FASTA	DNA	2342146	351836161	151	98.01	93.83	2342146	351404831	151	93.23	84.52	57	2345286	103	41145.4	648163	389368	300
CM2011-0629-0	FASTA	DNA	2096269	314735523	151	98.19	93.98	2096269	314391423	151	93.58	85.01	26	2324291	115	89395.8	753924	569265	271
CM2013-0017-0	FASTA	DNA	2191381	272492030	125	96.08	91.41	2191381	271959360	125	95.32	90.83	51	2311816	103	45329.7	453983	243874	236
CM2013-0823-1	FASTA	DNA	2336046	290549299	125	96.3	92.07	2336046	289915868	125	94.84	89.97	24	2221360	112	92556.7	527989	434580	261
CM2014-0491-0	FASTA	DNA	3712196	557211426	151	98.01	93.57	3712196	556537366	151	93.12	84.35	45	2248955	108	49976.8	611937	517085	495
CM2014-0657-0	FASTA	DNA	1885502	234519185	125	95.68	90.68	1885502	233993037	125	94.56	89.46	22	2258740	119	102670	555299	429324	207
CM2015-0068-1	FASTA	DNA	2256841	280714835	125	96.33	92.03	2256841	280009306	125	94.21	88.87	124	2282729	104	18409.1	168308	80024	246
CM2015-0090-1	FASTA	DNA	2181942	271385455	125	96.42	92.31	2181942	270809789	125	94.91	90.1	26	2393764	103	92067.8	618633	242156	227
CM2015-0350-0	FASTA	DNA	1891175	234819592	125	96.09	91.53	1891175	234061549	125	93.97	88.52	35	2245939	109	64169.7	608361	438224	209
CM2016-1071-0	FASTA	DNA	2954711	443626028	151	97.88	93.5	2954711	443119749	151	93.27	84.61	31	2273059	109	73324.5	524485	244607	390
CM2016-1071-1	FASTA	DNA	2852915	427986286	151	97.98	93.53	2852915	427467242	151	92.98	84.1	31	2272962	109	73321.4	524485	244607	376
CM2017-0508-0	FASTA	DNA	2175207	326334487	151	97.65	92.55	2175207	325976150	151	93.09	84.24	65	2335471	109	35930.3	598544	252448	279
CM2017-0740-5	FASTA	DNA	2680906	402294782	151	97.86	93.28	2680906	401857424	151	93.5	84.93	28	2308238	109	82437.1	622537	615116	348
CM2018-0104-0	FASTA	DNA	2090667	307958173	151	98.45	94.63	2090667	307700933	151	95.26	87.66	33	2341154	108	70944.1	615138	380725	263
CM2018-0111-0	FASTA	DNA	2458947	361757626	151	96.48	89.58	2458947	361557557	151	95.2	87.47	32	2341145	107	73160.8	622522	615138	309
CM2018-0111-1	FASTA	DNA	2774381	409192122	151	98.24	94.1	2774381	408822304	151	94.87	87.07	32	2341004	108	73156.4	622522	615138	349
CM2018-0364-0	FASTA	DNA	2500231	367953399	151	98	93.45	2500231	367681202	151	95.42	88.06	39	2263864	131	58047.8	602256	444554	325
CM2018-0476-1	FASTA	DNA	2503163	367543914	151	96.98	90.8	2503163	367351174	151	95.46	87.96	26	2239081	108	86118.5	611089	559710	328
CM2018-0643-0	FASTA	DNA	2146728	315691159	151	98.42	94.63	2146728	315439379	151	95.65	88.5	30	2350191	126	78339.7	609153	429563	269
CM2019-0106-0	FASTA	DNA	2204347	322924474	151	98.18	93.96	2204347	322607406	151	94.3	86.11	93	2221726	109	23889.5	171843	94824	291
CM2019-0177-0	FASTA	DNA	2720097	398820502	151	98.06	93.61	2720097	398309454	151	93.23	84.13	33	2263338	114	68586	422467	377807	352
CM2019-0327-0	FASTA	DNA	2415125	355057577	151	97.52	92.14	2415125	354859785	151	95.78	88.63	123	2274790	101	18494.2	171843	65310	312
CM2019-0508-0	FASTA	DNA	2761613	402264461	151	98.25	94.1	2761613	401977366	151	95.69	88.55	22	2293025	118	104228.4	602738	550608	351
CM2019-0543-0	FASTA	DNA	2902041	427922683	151	98.21	94.06	2902041	427498297	151	94.4	86.19	68	2540157	109	37355.3	505973	172503	337
CM2019-0603-0	FASTA	DNA	2867151	423816202	151	98.15	93.86	2867151	423313062	151	93.63	84.87	27	2339953	126	86664.9	554765	462226	362
CM2019-0691-0	FASTA	DNA	1941819	285802918	151	98.15	93.82	1941819	285531522	151	94.47	86.24	30	2199145	110	73304.8	605039	523220	260
CM2019-0691-1	FASTA	DNA	2453590	361986651	151	98.23	94.05	2453590	361650664	151	94.55	86.43	33	2199044	103	66637.7	565787	352384	329
CM2019-0692-0	FASTA	DNA	2121234	313041265	151	98.28	94.18	2121234	312752125	151	94.86	87.08	33	2199150	103	66640.9	565895	352384	285
CM2019-0891-0	FASTA	DNA	3038671	447341078	151	98.24	94.13	3038671	446929655	151	94.41	86.17	43	2297833	103	53438	598292	570124	389
CM2020-0075-0	FASTA	DNA	2145480	315782328	151	98.43	94.6	2145480	315506828	151	95.05	87.49	59	2327459	108	39448.5	646410	206427	271
CM2020-1034-0	FASTA	DNA	4165252	613094890	151	98.2	94.04	4165252	612486570	151	94.23	86.01	31	2289760	107	73863.2	577719	356502	535
CM2020-1144-0	FASTA	DNA	2715837	400307880	151	98.15	93.85	2715837	399921428	151	94.34	86.11	22	2288623	103	104028.3	559368	464938	350
CM2021-0098-0	FASTA	DNA	2278182	335387714	151	98.17	93.89	2278182	335111538	151	95.05	87.38	33	2326126	126	70488.7	468179	199820	288
CM2021-0114-0	FASTA	DNA	2326908	343071622	151	98.33	94.37	2326908	342717126	151	94.42	86.36	31	2297996	137	74128.9	768072	451390	298
CM2021-0118-0	FASTA	DNA	2188668	322362028	151	98.06	93.58	2188668	322054669	151	94.37	86.17	26	2318985	103	89191.7	559081	464927	278
CM2022-0178-1	FASTA	DNA	2734495	401209864	151	98.39	94.44	2734495	400953803	151	96.11	89.39	40	2253826	136	56345.7	514142	260938	356
CM2022-0178-2	FASTA	DNA	2728753	400229724	151	96.13	88.75	2728753	400044527	151	95.61	88.17	27	2255047	137	83520.3	613727	555050	355
CM2022-0184-0	FASTA	DNA	2373639	350703237	151	98.23	94.01	2373639	350394418	151	95.09	87.42	33	2370961	116	71847.3	622543	615138	296
CM2022-0265-1	FASTA	DNA	2430623	356625462	151	96.48	89.53	2430623	356469063	151	95.26	87.34	73	2307599	103	31610.9	604633	249909	309
CM2022-0265-2	FASTA	DNA	2261995	329373796	151	98	93.44	2261995	329095284	151	94.85	87.02	78	2276153	103	29181.4	604627	249909	289
CM2022-0430-0	FASTA	DNA	1457086	216630897	150	94.75	88.92	1457086	216490506	150	93.76	86.5	27	2197410	148	81385.6	595488	163302	197
CM2023-0122-0	FASTA	DNA	1397845	206838598	150	94.78	88.98	1397845	206726809	150	94.06	86.96	96	2381387	101	24806.1	369730	129868	174
CM2023-0137-0	FASTA	DNA	1563167	230228602	150	94.82	89.04	1563167	230122763	150	94.39	87.37	25	2251489	144	90059.6	598636	252596	204
CM2023-0222-2	FASTA	DNA	1633286	241108802	150	95.03	89.44	1633286	240978502	150	94.35	87.42	90	2331637	101	25907.1	252997	119013	207
CM2023-0226-0	FASTA	DNA	1544469	227718470	150	94.97	89.27	1544469	227593016	150	94.32	87.31	51	2260023	100	44314.2	534914	253105	201
CM2023-0265-0	FASTA	DNA	1516537	224505802	150	94.8	89.03	1516537	224348592	150	93.67	86.39	47	2360905	109	50232	381119	162517	190
CM2023-0277-0	FASTA	DNA	1534179	227369904	150	94.93	89.23	1534179	227237373	150	94.13	87.07	60	2391817	103	39863.6	250991	87946	190
