# Supplementary material for: Comparative genome analysis of Pasteurella multocida from Australian domestic animals suggests broad patterns of transmissions across multiple hosts and origins
Source: PLoS One. 2025 Aug 6;20(8):e0329807. doi: 10.1371/journal.pone.0329807 (PMC12327604; doi:10.1371/journal.pone.0329807)
Supplement: S4 Table — (PDF) [file pone.0329807.s004.pdf]

S4 Table. List of the 94 RefSeq genomes from the *Pasteurellaceae* family used to confirm taxonomic identification.

Accession	Species	Strain	Level of completion
GCF_001647695.1	[*Haemophilus*] *ducreyi*	VAN2	Complete
GCF_000007745.1	[*Mannheimia*] *succiniciproducens*	MBEL55E	Complete
GCF_000374285.1	*Actinobacillus capsulatus*	DSM 19761	Scaffold
GCF_900638385.1	*Actinobacillus delphinicola*	NCTC12871	Complete
GCF_000801145.1	*Actinobacillus equuli* subsp. *equuli*	19392	Complete
GCF_901764975.1	*Actinobacillus indolicus*	46K2C	Contig
GCF_900444945.1	*Actinobacillus lignieresii*	NCTC4189	Contig
GCF_000175195.1	*Actinobacillus minor*	NM305	Contig
GCF_900638445.1	*Actinobacillus pleuropneumoniae*	NCTC10976	Complete
GCF_901764995.1	*Actinobacillus porcinus*	NM319	Scaffold
GCF_003101015.1	*Actinobacillus porcitonsillarum*	9953L55	Complete
GCF_900460625.1	*Actinobacillus seminis*	NCTC10851	Contig
GCF_002591855.1	*Actinobacillus succinogenes*	GXAS137	Scaffold
GCF_000739435.1	*Actinobacillus suis*	ATCC 33415	Complete
GCF_900445015.1	*Actinobacillus ureae*	NCTC10220	Contig
GCF_901687125.1	*Actinobacillus vicugnae*	W16181	Contig
GCF_023518055.1	*Aggregatibacter actinomycetemcomitans*	4S	Complete
GCF_900636915.1	*Aggregatibacter aphrophilus*	NCTC 5906	Complete
GCF_003130255.1	*Aggregatibacter kilianii*	PN_528	Contig
GCF_900476035.1	*Aggregatibacter segnis*	NCTC 10977	Complete
GCF_900454535.1	*Avibacterium avium*	NCTC 11297	Contig
GCF_002921145.1	*Avibacterium endocarditidis*	20186H4H1	Scaffold
GCF_004362535.1	*Avibacterium gallinarum*	DSM 17481	Scaffold
GCF_011765605.1	*Avibacterium paragallinarum*	ESV-135	Complete
GCF_900635775.1	*Avibacterium volantium*	NCTC3438	Complete
GCF_011455875.1	*Basfia succiniciproducens*	JF4016	Complete
GCF_000521725.1	*Bibersteinia trehalosi*	USDA-ARS-USMARC-188	Complete
GCF_013377195.1	*Bisgaardia hudsonensis*	M327/99/2	Complete
GCF_900450725.1	*Canicola haemoglobinophilus*	NCTC1659	Contig
GCF_002795405.1	*Caviibacterium pharyngocola*	7.3	Contig
GCF_000772535.1	*Chelonobacter oris*	1662	Contig
GCF_002795425.1	*Conservatibacter flavescens*	7.4	Contig
GCF_004340985.1	*Cricetibacter osteomyelitidis*	DSM 28404	Contig
GCF_011455495.1	*Frederiksenia canicola*	HPA 21	Complete
GCF_000772265.1	*Gallibacterium anatis*	F149	Contig
GCF_001678495.1	*Gallibacterium salpingitidis*	F150	Contig
GCF_003260095.1	*Glaesserella australis*	HS4635	Scaffold
GCF_017352235.1	*Glaesserella parasuis*	YHP1818	Complete
GCF_900475885.1	*Haemophilus aegyptius*	NCTC8502	Complete
GCF_900477945.1	*Haemophilus haemolyticus*	NCTC10839	Complete
GCF_000931575.1	*Haemophilus influenzae*	477	Complete
GCF_002015115.1	*Haemophilus paracuniculus*	CCUG 43573	Scaffold
GCF_016889385.1	*Haemophilus parahaemolyticus*	FDAARGOS_1199	Complete
GCF_000191405.1	*Haemophilus parainfluenzae*	ATCC 33392	Scaffold
GCF_900451065.1	*Haemophilus paraphrohaemolyticus*	NCTC10671	Contig
GCF_900186995.1	*Haemophilus pittmaniae*	NCTC13334	Complete
GCF_008605885.1	*Haemophilus seminalis*	SZY H2	Scaffold
GCF_000287615.1	*Haemophilus sputorum*	HK 2154	Contig
GCF_000019405.1	*Histophilus somni*	2336	Complete
GCF_004339625.1	*Lonepinella koalarum*	DSM 10053	Scaffold
GCF_014541205.1	*Mannheimia bovis*	ZY190616	Complete
GCF_011455695.1	*Mannheimia granulomatis*	B 234/94	Complete
GCF_002285575.1	*Mannheimia haemolytica*	USDA-ARS-USMARC-191	Complete
GCF_000940515.1	*Mannheimia massilioguelmaensis*	MG13T	Contig
GCF_009828705.1	*Mannheimia ovis*	ZY170218	Complete

GCF_013378015.1	*Mannheimia pernigra*	16CN0041	Complete
GCF_000521695.1	*Mannheimia varigena*	USDA-ARS-USMARC-1312	Complete
GCF_003265225.1	*Mergibacter septicus*	27517-4-I1	Complete
GCF_004363295.1	*Mesocricetibacter intestinalis*	DSM 28403	Scaffold
GCF_004569585.1	*Muribacter muris*	WT12	Contig
GCF_000827595.2	*Necropsobacter massiliensis*	FF6	Scaffold
GCF_004345745.1	*Nicoletella semolina*	DSM 16380	Scaffold
GCF_009684715.1	*Otariodibacter oris*	Baika1	Complete
GCF_018343795.1	*Pasteurella atlantica*	NVI-9100	Complete
GCF_000262245.1	*Pasteurella bettyae*	CCUG 2042	Contig
GCF_024622045.1	*Pasteurella caecimuris*	DSM 28627	Contig
GCF_020810675.1	*Pasteurella canis*	HL_NV12211	Complete
GCF_900186835.1	*Pasteurella dagmatis*	NCTC11617	Complete
GCF_003096995.1	*Pasteurella langaaensis*	DSM 22999	Contig
GCF_002073255.2	*Pasteurella multocida*	FDAARGOS_218	Complete
GCF_002850605.1	*Pasteurella oralis*	WCHPO000540	Contig
GCF_013377295.1	*Pasteurella skyensis*	95A1	Complete
GCF_900454705.1	*Pasteurella testudinis*	NCTC12150	Contig
GCF_900454895.1	*Phocoenobacter uteri*	NCTC12872	Contig
GCF_003585965.1	*Psittacicella gerlachiana*	EEAB3T1	Contig
GCF_003585935.1	*Psittacicella hinzii*	111	Contig
GCF_003585925.1	*Psittacicella melopsittaci*	B96_4	Contig
GCF_015356115.1	*Rodentibacter haemolyticus*	DSM 111151	Complete Genome
GCF_002000125.1	*Rodentibacter heidelbergensis*	Ac69	Contig
GCF_010587025.1	*Rodentibacter heylii*	G1	Complete Genome
GCF_001998825.1	*Rodentibacter mrazii*	Ppn418	Contig
GCF_001999305.1	*Rodentibacter myodis*	Ac151	Contig
GCF_000730685.1	*Rodentibacter pneumotropicus*	ATCC 35149	Contig
GCF_001998965.1	*Rodentibacter rarus*	CCUG 17206	Contig
GCF_002000485.1	*Rodentibacter ratti*	F75	Contig
GCF_002000425.1	*Rodentibacter trehalosifermentans*	H1983213011	Contig
GCF_014885015.1	*Spirabiliibacterium falconis*	NCTC 11878	Contig
GCF_014884965.1	*Spirabiliibacterium mucosae*	20609_3	Contig
GCF_014884995.1	*Spirabiliibacterium pneumoniae*	HPA106	Contig
GCF_004342725.1	*Testudinibacter aquarius*	DSM 28140	Scaffold
GCF_009761375.1	*Ursidibacter arcticus*	Bamse1	Contig
GCF_009761395.1	*Ursidibacter maritimus*	Pb43106	Contig
GCF_013377275.1	*Vespertiliibacter pulmonis*	CCUG 64585	Chromosome
GCF_004339025.1	*Volucribacter psittacicida*	DSM 15534	Scaffold
