# Supplementary material for: Comparative genome analysis of Pasteurella multocida from Australian domestic animals suggests broad patterns of transmissions across multiple hosts and origins
Source: PLoS One. 2025 Aug 6;20(8):e0329807. doi: 10.1371/journal.pone.0329807 (PMC12327604; doi:10.1371/journal.pone.0329807)
Supplement: S5 Table — (PDF) [file pone.0329807.s005.pdf]

S5 Table. Country and host distribution of whole genome sequences of *P. multocida* analysed with PopPUNK.

Data Source	Country	Domestic Bird	Ruminant	Pig	Rabbit	Dog or Cat	Human	Wildlife	Not specified	Horse	Environment	# genomic sequences by country & data source
GenBank	Australia	23	4	1		14	22	3				67
	Bangladesh	8	1									9
	Canada		1									1
	China	18	12	58	26		1	5	13			133
	France				17							17
	Germany		1	3								4
	Greece					1	1					2
	India		25	3								28
	Iran		1									1
	Japan	1							1			2
	Kazakhstan		1							2		3
	Malaysia						1		1			2
	Morocco		1									1
	Myanmar		1									1
	Netherlands						1					1
	New Zealand								4			4
	Not specified	30	13	5	18		4	3	18			91
	Pakistan		11						1			12
	Peru		8					1				9
	Russia		5	1					1			7
	South Korea					1						1
	Spain		14				2					16
	Sri Lanka		1									1
	Switzerland		1									1
	Thailand		3									3
	United Kingdom		14	4								18
	USA	22	57			1	4				2	86
	Vietnam	1										1
	Tunisia						1					1
SRA	Australia	148						14				162
This study	Australia	18	10	1	5	20		4			1	59
# genomic sequences by host		270	185	76	66	37	37	30	39	2	3	744
