# Supplementary material for: Comparative genome analysis of Pasteurella multocida from Australian domestic animals suggests broad patterns of transmissions across multiple hosts and origins
Source: PLoS One. 2025 Aug 6;20(8):e0329807. doi: 10.1371/journal.pone.0329807 (PMC12327604; doi:10.1371/journal.pone.0329807)
Supplement: S6 Table — Refer to Table 2 for the five subsets (A-E) containing mixed host species. (PDF) [file pone.0329807.s006.pdf]

S6 Table. GoeBURST groups with less than 4 loci differences. Refer to Table 2 for the five subsets (A-E) containing mixed host species.

goeBURST MST[4]	Genome	Strain	Host	Country	MLST (RIRDC)
1	GCF_001929595.1_ASM192959v1	2450PM	Ruminant	USA	79
	GCF_001929655.1_ASM192965v1	2512PM	Ruminant	USA	79
	GCF_001929705.1_ASM192970v1	2578PM	Ruminant	USA	79
	GCF_001929765.1_ASM192976v1	2633PM	Ruminant	USA	79
	GCF_001929785.1_ASM192978v1	2612PM	Ruminant	USA	79
	GCF_001929855.1_ASM192985v1	2930PM	Ruminant	USA	79
	GCF_001929905.1_ASM192990v1	2125PM	Ruminant	USA	79
	GCF_001929985.1_ASM192998v1	2165PM	Ruminant	USA	79
	GCF_001930065.1_ASM193006v1	2267PM	Ruminant	USA	79
	GCF_001930115.1_ASM193011v1	2297PM	Ruminant	USA	79
	GCF_001930285.1_ASM193028v1	2389PM	Ruminant	USA	79
	GCF_001930305.1_ASM193030v1	2335PM	Ruminant	USA	79
	GCF_001930405.1_ASM193040v1	2497PM	Ruminant	USA	79
	GCF_001930465.1_ASM193046v1	2597PM	Ruminant	USA	79
	GCF_001930525.1_ASM193052v1	2668PM	Ruminant	USA	79
	GCF_001930765.1_ASM193076v1	3045PM	Ruminant	USA	79
	GCF_001930875.1_ASM193087v1	3275PM	Ruminant	USA	79
	GCF_001930925.1_ASM193092v1	3384PM	Ruminant	USA	79
	GCF_001931145.1_ASM193114v1	69APM	Ruminant	USA	79
	GCF_001931235.1_ASM193123v1	2901PM	Ruminant	USA	79
2 (A)	GCF_035520835.1	Pm1620	Dog/Cat	Australia	20
	GCF_035520915.1	Pm1613	Dog/Cat	Australia	20
	GCF_035521535.1	Pm1612	Dog/Cat	Australia	20
	U13-0203-0	CM2013-0203-0	Wildlife	Australia	20
	U13-0425-0	CM2013-0425-0	Wildlife	Australia	20
	U17-0740-0	CM2017-0740-0	Bird	Australia	20
	U17-0740-1	CM2017-0740-1	Bird	Australia	20
	U17-0740-2	CM2017-0740-2	Bird	Australia	20
	U22-0138-0	CM2022-0138-0	Bird	Australia	20
3 (B)	GCF_000296345.2_ASM29634v2	VTCCBAA264	Ruminant	India	122
	GCF_001029495.1_ASM102949v1	BUKK	Ruminant	Pakistan	122
	GCF_003268295.1_ASM326829v1	BAUTB2	Ruminant	Bangladesh	122
	GCF_014338445.1_ASM1433844v1	Ban-PM4	Bird	Bangladesh	122
	GCF_014338465.1_ASM1433846v1	Ban-PM7	Bird	Bangladesh	122
	GCF_022213185.1_ASM2221318v1	PmBUFF2016HRY	Ruminant	India	122
	GCF_022575965.1_ASM2257596v1	DC2020	Bird	Bangladesh	122
	GCF_022869075.1_ASM2286907v1	NIVEDIPm35	Ruminant	India	122
	GCF_026738975.1_ASM2673897v1	Alim_FC_1002	Bird	Bangladesh	122
	GCF_026739035.1_ASM2673903v1	Alim_FC_1000	Bird	Bangladesh	122
	GCF_026739075.1_ASM2673907v1	Alim_FC_1001	Bird	Bangladesh	122
	GCF_028826065.1_ASM2882606v1	Alim_FC_1003	Bird	Bangladesh	122
4	GCF_001929565.1_ASM192956v1	2403PM	Ruminant	USA	79
	GCF_001930385.1_ASM193038v1	2428PM	Ruminant	USA	79
	GCF_001930445.1_ASM193044v1	2526PM	Ruminant	USA	79
	GCF_001930605.1_ASM193060v1	2887PM	Ruminant	USA	79
	GCF_001930895.1_ASM193089v1	3347PM	Ruminant	USA	79
	GCF_001931265.1_ASM193126v1	2969PM	Ruminant	USA	79
5	U14-0810-0	CM2014-0810-0	Bird	Australia	20
	U16-1019-2	CM2016-1019-2	Bird	Australia	20
	U16-1054-3	CM2016-1054-3	Bird	Australia	20
	U16-1054-4	CM2016-1054-4	Bird	Australia	20
	U16-1086-0	CM2016-1086-0	Bird	Australia	20
	U16-1086-1	CM2016-1086-1	Bird	Australia	20
	U17-0502-0	CM2017-0502-0	Bird	Australia	20
	U17-0502-1	CM2017-0502-1	Bird	Australia	20

	U17-0740-3	CM2017-0740-3	Bird	Australia	20
	U17-0740-4	CM2017-0740-4	Bird	Australia	20
	U17-1139-0	CM2017-1139-0	Bird	Australia	20
	U17-1139-1	CM2017-1139-1	Bird	Australia	20
6	GCF_001670535.1_ASM167053v1	ATCC_2095	Bird	-	159
	GCF_023556035.1_ASM2355603v1	P2095	Bird	-	159
	GCF_023573065.1_ASM2357306v1	P2095	Bird	USA	159
7	GCF_035520695.1	Pmc-B	Dog/Cat	Australia	30
	GCF_035520755.1	Pmc-A	Dog/Cat	Australia	30
	GCF_035520795.1	Pmc-C	Dog/Cat	Australia	30
8	GCF_001027665.1_ASM102766v1	PVAcc	-	Pakistan	122
	GCF_001027685.1_ASM102768v1	V1	Ruminant	Pakistan	122
	GCF_001027735.1_ASM102773v1	Islm	Ruminant	Pakistan	122
	GCF_001027805.1_ASM102780v1	Pesh	Ruminant	Pakistan	122
	GCF_001028205.1_ASM102820v1	Faisal	Ruminant	Pakistan	122
	GCF_001028225.1_ASM102822v1	ATTK	Ruminant	Pakistan	122
	GCF_001028245.1_ASM102824v1	Karachi	Ruminant	Pakistan	122
	GCF_004114645.1_ASM411464v1	PM3	Ruminant	Pakistan	122
	GCF_016458265.1_ASM1645826v1	PM1	Ruminant	Pakistan	122
	GCF_016916875.1_ASM1691687v1	PM2	Ruminant	Pakistan	122
9	GCF_029873295.1_ASM2987329v1	19BRD-057	Ruminant	Australia	394
	GCF_029906265.1_ASM2990626v1	19BRD-032	Ruminant	Australia	394
	GCF_029906285.1_ASM2990628v1	18BRD-001	Ruminant	Australia	394
	GCF_032027925.1_ASM3202792v1	17BRD-035	Ruminant	Australia	394
10	GCF_002023805.1_ASM202380v1	CIRMBP-0760	Rabbit	France	298
	GCF_002023825.1_ASM202382v1	CIRMBP-0758	Rabbit	France	298
	GCF_023555595.1_ASM2355559v1	B615	Rabbit	-	298
11	GCF_001931225.1_ASM193122v1	8522APM	Ruminant	USA	79
	GCF_002859385.1_ASM285938v1	USDA-ARS-USMARC-60224	Ruminant	USA	79
	GCF_002859485.1_ASM285948v1	USDA-ARS-USMARC-59962	Ruminant	USA	79
12	GCF_001931155.1_ASM193115v1	654BPM	Ruminant	USA	79
	GCF_001931185.1_ASM193118v1	657BPM	Ruminant	USA	79
	GCF_001931455.1_ASM193145v1	J1APM	Ruminant	USA	79
13	GCF_000298655.1_X73v1	X73	-	-	60
	GCF_002073295.2_ASM207329v2	FDAARGOS_216	Bird	USA	60
14	GCF_002083265.2_ASM208326v2	FDAARGOS_217	Ruminant	USA	122
	GCF_023555995.1_ASM2355599v1	M1404	Ruminant	-	122
15	GCF_023572885.1_ASM2357288v1	10185	Bird	USA	235
	GCF_023572985.1_ASM2357298v1	11245	Bird	USA	235
16	GCF_035221255.1	Pm1618	Dog/Cat	Australia	30
	GCF_035520775.1	Pm1622	Dog/Cat	Australia	30
17	GCF_036348715.1	B_OL	Ruminant	Russia	122
	GCF_036348755.1	B_Kr	Ruminant	Russia	122
18	U18-0104-0	CM2018-0104-0	Bird	Australia	8
	U18-0111-0	CM2018-0111-0	Bird	Australia	8
	U18-0111-1	CM2018-0111-1	Bird	Australia	8
	U22-0184-0	CM2022-0184-0	Bird	Australia	8
19	GCF_016313205.1_ASM1631320v1	PM-1	Ruminant	China	122
	GCF_031432935.1_ASM3143293v1	sample-B	Ruminant	China	122
20	GCF_009663815.1_ASM966381v1	P52	Ruminant	India	122
	GCF_022213195.1_ASM2221319v1	Pm52HVVI	Ruminant	India	122
21	GCF_001857595.1_ASM185759v1	HS_SKN01	Ruminant	India	122
	GCF_022869035.1_ASM2286903v1	NIVEDIPm32	Ruminant	India	122
	GCF_022869065.1_ASM2286906v1	NIVEDIPm34	Ruminant	India	122
22	SRR12130735	PM1582	Bird	Australia	451
	SRR12130737	PM1541	Bird	Australia	451
	SRR12130738	PM1447	Bird	Australia	451
23	U17-0508-0	CM2017-0508-0	Environment	Australia	3
	U19-0891-0	CM2019-0891-0	Dog/Cat	Australia	3

24	GCF_013013425.1_ASM1301342v1	HND12	Pig	China	287
	GCF_013013505.1_ASM1301350v1	HND13	Pig	China	287
25 (C)	GCF_001661585.1_ASM166158v1	HB02	Bird (Duck)	China	129
	GCF_001662525.1_C48_V1	C48-1	Bird (Chicken)	China	129
	GCF_004286945.1_ASM428694v1	C48-1	Bird (Chicken)	China	129
26	GCF_013013655.1_ASM1301365v1	HNF02	Pig	China	9
	GCF_013013685.1_ASM1301368v1	HNF01	Pig	China	9
27	U22-0189-1	CM2022-0189-1	Bird	Australia	20
	U22-0189-2	CM2022-0189-2	Bird	Australia	20
28	GCF_026315085.1_ASM2631508v1	PF6	Rabbit	China	428
	GCF_026315145.1_ASM2631514v1	PF9	Rabbit	China	428
	GCF_026315165.1_ASM2631516v1	PF10	Rabbit	China	428
	GCF_026409225.1_ASM2640922v1	PF12	Rabbit	China	428
	GCF_026409265.1_ASM2640926v1	PF16	Rabbit	China	428
	GCF_026409305.1_ASM2640930v1	PF18	Rabbit	China	428
	GCF_026409325.1_ASM2640932v1	PF1	Rabbit	China	428
29	GCF_002023985.1_ASM202398v1	CIRMBP-0922	Rabbit	France	9
	GCF_002024065.1_ASM202406v1	CIRMBP-0747	Rabbit	France	9
30	GCF_004286965.1_ASM428696v1	Pm72-4	Bird	China	-
	GCF_004286975.1_ASM428697v1	Pm731	Bird	-	129
31	GCF_019134675.1_ASM1913467v1	PMWSG-4	Bird	China	129
	GCF_029762475.1_ASM2976247v1	Pm-3	-	China	129
32	GCF_001929465.1_ASM192946v1	2154PM	Ruminant	USA	79
	GCF_001929525.1_ASM192952v1	2320PM	Ruminant	USA	79
33	GCF_003402875.1_ASM340287v1	PM8-1	Pig	China	13
	GCF_003402895.1_ASM340289v1	TB168	-	China	13
	GCF_003402915.1_ASM340291v1	EB168	-	China	13
	GCF_006351865.1_ASM635186v1	PM_8-6	Pig	China	13
34	GCF_027951085.1_ASM2795108v1	NIVEDIPm20	Ruminant	India	288
	GCF_027951095.1_ASM2795109v1	NIVEDIPm3	Ruminant	India	288
	GCF_027951115.1_ASM2795111v1	NIVEDIPm1	Ruminant	India	288
35	GCF_033795715.1_ASM3379571v1	720CV	Ruminant	Spain	79
	GCF_033795845.1_ASM3379584v1	189CV	Ruminant	Spain	79
36	GCF_023556285.1_ASM2355628v1	P1933	Ruminant	-	80
	GCF_030846735.1_ASM3084673v1	P1933	Ruminant	USA	80
37	GCF_033795675.1_ASM3379567v1	756CM	Ruminant	Spain	13
	GCF_033795785.1_ASM3379578v1	432PM	Ruminant	Spain	13
38	GCF_002859265.1_ASM285926v1	USDA-ARS-USMARC-60215	Ruminant	USA	79
	GCF_002859325.1_ASM285932v1	USDA-ARS-USMARC-59910	Ruminant	USA	79
	GCF_002859525.1_ASM285952v1	USDA-ARS-USMARC-60381	Ruminant	USA	79
39 (D)	GCF_029324765.1_ASM2932476v1	1231	Pig	Russia	74
	GCF_029324775.1_ASM2932477v1	T-80	Ruminant	Russia	74
40	GCF_001670555.1_ASM167055v1	ATCC_2100	Bird	-	27
	GCF_024055495.1_ASM2405549v1	P2100	Bird	USA	27
41	GCF_014058465.1_ASM1405846v1	A0419	Ruminant	United_Kingdom	349
	GCF_014058485.1_ASM1405848v1	A0757	Ruminant	United_Kingdom	349
	GCF_014058585.1_ASM1405858v1	X0120	Ruminant	United_Kingdom	349
	GCF_014058605.1_ASM1405860v1	X1053	Ruminant	United_Kingdom	349
	GCF_014058625.1_ASM1405862v1	618/90	Ruminant	United_Kingdom	349
	GCF_014058645.1_ASM1405864v1	619/90	Ruminant	United_Kingdom	349
	GCF_014058665.1_ASM1405866v1	671/90	Ruminant	United_Kingdom	349
42	GCF_026153335.1_ASM2615333v1	P5041881	Pig	United_Kingdom	50
	GCF_026153375.1_ASM2615337v1	P504190	Pig	United_Kingdom	50
43	GCF_013013235.1_ASM1301323v1	HND01	Pig	China	50
	GCF_013013455.1_ASM1301345v1	HND10	Pig	China	50
44	GCF_023587085.1_ASM2358708v1	21317	Bird	-	481
	GCF_023587205.1_ASM2358720v1	35564	Bird	-	481
45	GCF_002083215.2_ASM208321v2	FDAARGOS_220	Environment	USA	156
	GCF_023556255.1_ASM2355625v1	P1702	Bird	-	156

46	GCF_012952165.1_ASM1295216v1	92_67_2	Bird	USA	8
	GCF_012952225.1_ASM1295222v1	93_182	Bird	USA	8
47	GCF_000298675.1_P1059v1	P1059	Bird	USA	8
	GCF_001670775.1_ASM167077v1	ATCC_15742	Bird	-	8
	GCF_002073255.2_ASM207325v2	FDAARGOS_218	Environment	USA	8
48	U22-0178-1	CM2022-0178-1	Rabbit	Australia	25
	U22-0178-2	CM2022-0178-2	Rabbit	Australia	25
49	GCF_012271895.1_ASM1227189v1	P-mult-15-KZ	Horse	Kazakhstan	460
	GCF_012271915.1_ASM1227191v1	P-mult-5-KZ	Horse	Kazakhstan	460
50	GCF_023555855.1_ASM2355585v1	VP243	Ruminant	-	162
	GCF_023555895.1_ASM2355589v1	VP584	Ruminant	-	162
51	U19-0691-0	CM2019-0691-0	Bird	Australia	9
	U19-0691-1	CM2019-0691-1	Bird	Australia	9
	U19-0692-0	CM2019-0692-0	Bird	Australia	9
52	GCF_002393385.1_ASM239338v1	FDAARGOS_384	Human	USA	25
	GCF_002591295.1_ASM259129v1	FDAARGOS_385	Human	USA	25
53	GCF_035220555.1	Pm1621	Dog/Cat	Australia	265
	GCF_035520875.1	Pm1617	Dog/Cat	Australia	265
	GCF_035521575.1	Pm1616	Dog/Cat	Australia	265
54	GCF_000731735.1_ASM73173v1	2213	Ruminant	India	122
	GCF_000731745.1_ASM73174v1	3213	Ruminant	India	122
55	GCF_900115485.1	ATCC_51689	-	-	65
	GCF_900636625.1_43295_B02	NCTC10204	Ruminant	United_Kingdom	65
56	GCF_002023835.1_ASM202383v1	CIRMBP-0812	Rabbit	France	204
	GCF_002023845.1_ASM202384v1	CIRMBP-0749	Rabbit	France	204
57	GCF_026315065.1_ASM2631506v1	PF5	Rabbit	China	430
	GCF_026315125.1_ASM2631512v1	PF7	Rabbit	China	430
	GCF_026636255.1_ASM2663625v1	PF8	Rabbit	China	430
58 (E)	GCF_002930755.1_ASM293075v1	S298D	Dog/Cat	Greece	4
	GCF_002930775.1_ASM293077v1	PY81579	Human	Greece	4
59	U21-0180-0	CM2021-0180-0	Bird	Australia	20
	U21-0180-1	CM2021-0180-1	Bird	Australia	20
	U21-0180-2	CM2021-0180-2	Bird	Australia	20
60	GCF_026315225.1_ASM2631522v1	PF11	Rabbit	China	430
	GCF_026409285.1_ASM2640928v1	PF19	Rabbit	China	430
	GCF_026409345.1_ASM2640934v1	PF17	Rabbit	China	430
61	U23-0122-0	CM2023-0122-0	Ruminant	Australia	394
	U23-0222-2	CM2023-0222-2	Ruminant	Australia	394
62	GCF_036688525.1	PM147	-	-	129
	GCF_036688545.1	PM115	-	-	129
	GCF_036688585.1	PM148	-	-	129
	GCF_036688605.1	PM114	-	-	129
	GCF_036688635.1	PM02	-	-	129
	GCF_036688655.1	PM03	-	-	129
63	GCF_026409365.1_ASM2640936v1	PF15	Rabbit	China	431
	GCF_026723845.1_ASM2672384v1	PF13	Rabbit	China	431
64	GCF_001670425.2_ASM167042v2	HN141014	Bird	China	129
	GCF_001670445.1_ASM167044v1	DY120818	Bird	China	129
65	GCF_002859245.1_ASM285924v1	USDA-ARS-USMARC-60494	Ruminant	USA	79
	GCF_002859545.1_ASM285954v1	USDA-ARS-USMARC-60714	Ruminant	USA	79
66	U16-1071-0	CM2016-1071-0	Ruminant	Australia	79
	U16-1071-1	CM2016-1071-1	Ruminant	Australia	79
67	GCF_000754275.1_ASM75427v1	ATCC_43137	-	-	13
	GCF_900187275.1_51765_F01	NCTC10322	Pig	-	13
68	GCF_013013075.1_ASM1301307v1	HNA14	Pig	China	13
	GCF_013013125.1_ASM1301312v1	HNA15	Pig	China	13
69	GCF_013013165.1_ASM1301316v1	HNA19	Pig	China	13
	GCF_013013175.1_ASM1301317v1	HNA18	Pig	China	13
	GCF_013013205.1_ASM1301320v1	HNA20	Pig	China	13

70	GCF_002859365.1_ASM285936v1	USDA-ARS-USMARC-60713	Ruminant	USA	79
	GCF_002859405.1_ASM285940v1	USDA-ARS-USMARC-60712	Ruminant	USA	79
71	GCF_002859285.1_ASM285928v1	USDA-ARS-USMARC-60717	Ruminant	USA	79
	GCF_002859305.1_ASM285930v1	USDA-ARS-USMARC-60213	Ruminant	USA	79
72	GCF_001874105.1_ASM187410v1	unmsm	Ruminant	Peru	321
	GCF_002900115.1_ASM290011v1	UNMSM2	Ruminant	Peru	321
	GCF_015354635.1_ASM1535463v1	PMUVET4	Ruminant	Peru	321
	GCF_015354645.1_ASM1535464v1	PMUVET5	Ruminant	Peru	321
	GCF_015354675.1_ASM1535467v1	PMUVET3	Ruminant	Peru	321
	GCF_015354685.1_ASM1535468v1	PMUVET2	Ruminant	Peru	321
	GCF_015354715.1_ASM1535471v1	PMUVET1	Ruminant	Peru	321
	GCF_028437575.1_ASM2843757v1	PM2	Ruminant	Peru	321
73	GCF_002859465.1_ASM285946v1	USDA-ARS-USMARC-60385	Ruminant	USA	80
	GCF_002859505.1_ASM285950v1	USDA-ARS-USMARC-60214	Ruminant	USA	-
74	GCF_027951105.1_ASM2795110v1	NIVEDIPm9	Ruminant	India	288
	GCF_027951325.1_ASM2795132v1	NIVEDIPm10	Ruminant	India	288
75	GCF_030059805.1_ASM3005980v1	P030653/2	Pig	United_Kingdom	50
	GCF_030122645.1_ASM3012264v1	P030653/1	Pig	United_Kingdom	50
76	GCF_002750835.1_ASM275083v1	SH02	Pig	China	457
	GCF_002817545.1_ASM281754v1	SH01	Pig	China	457
