# Supplementary material for: Comparative genome analysis of Pasteurella multocida from Australian domestic animals suggests broad patterns of transmissions across multiple hosts and origins
Source: PLoS One. 2025 Aug 6;20(8):e0329807. doi: 10.1371/journal.pone.0329807 (PMC12327604; doi:10.1371/journal.pone.0329807)
Supplement: Attachment — Submitted filename: Allen et all Plos One 2024 Review.docx [file pone.0329807.s007.docx]

The manuscript presents a genomic and phylogenetic analysis of Pasteurella multocida isolates, with a focus on Australian companion animals. It is a well-written study that significantly contributes to understanding the genomic diversity of this pathogen. However, it overlooks several key publications, particularly recent genomic studies from Australia. While I understand that some of these publications on P. multocida from human and companion animals were released after the authors' NCBI GenBank access date (December 13, 2023), it is unfortunate that they will need to redo portions of their analysis. As their work focuses on Australia, incorporating this new data would strengthen the manuscript's relevance and provide a more comprehensive discussion of the pathogen in this region. Below, I provide a detailed review with recommendations to include the new genomic data, improve clarity, and refine specific analyses.

The manuscript claims there were no genome from Australian companion animals at the date of NCBI GenBank database access. However, genomes of several P. *multocida* isolates from human and companion animals from Australia have been analysed and deposited in public database. Given the manuscript’s focus on Australian isolates, I recommend incorporating this recent data to update the results and discussion.

Specifically, the following publication available from March 1, 2024 could significantly inform the current analysis:

Smallman TR, Perlaza-Jiménez L, Wang X, Korman TM, Kotsanas D, Gibson JS, et al. Pathogenomic analysis and characterization of *Pasteurella multocida* strains recovered from human infections. Microbiol Spectr. 2024;12(4):e0380523. (available from 1^st^ of March 2024)

Furthermore, the authors may want to consider incorporating data from a recently published complete genome of a P. multocida isolate from an Australian pig

Chau D, Blackall PJ, Turni C, Omaleki L. Complete genome sequence of a *Pasteurella multocida* isolate from a pig in Australia. Microbiology resource announcements.0(0):e00241-24.

Result section:

Figures 2 and 3: The phylogenetic trees are similar to those described in the Smallman et al. publication, with a distinct deep-branched cluster. However, the quality of the images in this manuscript made it difficult to access the phylogenetic relationships. To improve the clarity and robustness of the phylogenetic analysis, I suggest comparing these results with those from Smallman et al., which may offer additional insights and reinforce the authors' conclusions.

Line 255: The manuscript suggests that close phylogenetic relatedness and identical MLST profiles indicate host transfers. While this is possible, it is not definitive. Tools such as PHYLOViZ could be used to more accurately represent evolutionary relationships between strains. For example, the chicken isolate (CM2007-0542-0) and duck isolate (CM2013-0823-1) share a common ancestor but this does not necessarily imply direct transfer from one host to the other.

Line 260: The manuscripts states that Australian isolates PM1541 and PM1582 belong to ST451. However, according to the original publication, these isolates were identified as ST274 (both based on RIRDC scheme). I suggest the authors revisit the data to confirm the correct sequence type.

Line 262: The Smallman TR et al., publication also demonstrates the clustering of the USA strain FDAARGOS_384 and an Australian human isolate. Comparing the findings from these two manuscripts would provide further insight into global strain distribution and zoonotic transmission patterns.

Line 317: The observation that subspecies classification in published genomes does not match phylogeny closely is an important point. To explore this further, I recommend an *in-silico* approach using available genomic data to differentiate between subspecies. Ujvári et al. (2022) provide a framework for identifying key genes associated with *Pasteurella multocida* subspecies, which could be useful for this reanalysis. This method would enable verification of the GenBank metadata and address potential misclassifications without the need for additional laboratory work.

Ujvári, B., Gantelet, H., Magyar, T., 2022. Development of a multiplex PCR assay for the detection of key genes associated with *Pasteurella multocida* subspecies. J. Vet. Diagn. Invest. 34, 319-322.

I also suggest incorporating findings from Alhamami et al. (2023), which focus on antimicrobial resistance genes (ARG) in *P. multocida* isolates from Australian feedlot cattle. This would enhance the discussion around ARG in *P. multocida* originated from various host species.

Alhamami T, Roy Chowdhury P, Venter H, Veltman T, Truswell A, Abraham S, et al. Genomic profiling of Pasteurella multocida isolated from feedlot cases of bovine respiratory disease. Vet Microbiol. 2023;283:109773.
