# Supplementary material for: Comparative genome analysis of Pasteurella multocida from Australian domestic animals suggests broad patterns of transmissions across multiple hosts and origins
Source: PLoS One. 2025 Aug 6;20(8):e0329807. doi: 10.1371/journal.pone.0329807 (PMC12327604; doi:10.1371/journal.pone.0329807)
Supplement: Attachment — Submitted filename: Response to reviewers.docx [file pone.0329807.s010.docx]

Dear PLOS ONE Editors,

Thank you for providing us with the opportunity to submit a revised version of our manuscript.

PONE-D-24-33400

Comparative genome analysis of *Pasteurella multocida* from Australian domestic animals suggests broad patterns of transmissions across multiple hosts and origins

Please submit your revised manuscript by Oct 30 2024 11:59PM. If you will need more time than this to complete your revisions, please reply to this message or contact the journal office at [plosone@plos.org](mailto:plosone@plos.org). When you're ready to submit your revision, log on to https://www.editorialmanager.com/pone/ and select the 'Submissions Needing Revision' folder to locate your manuscript file.

Please include the following items when submitting your revised manuscript:

- A rebuttal letter that responds to each point raised by the academic editor and reviewer(s). You should upload this letter as a separate file labeled 'Response to Reviewers'.
- A marked-up copy of your manuscript that highlights changes made to the original version. You should upload this as a separate file labeled 'Revised Manuscript with Track Changes'.
- An unmarked version of your revised paper without tracked changes. You should upload this as a separate file labeled 'Manuscript'.

Journal requirements:

When submitting your revision, we need you to address these additional requirements.

1. Please ensure that your manuscript meets PLOS ONE's style requirements, including those for file naming. The PLOS ONE style templates can be found at

<https://journals.plos.org/plosone/s/file?id=wjVg/PLOSOne_formatting_sample_main_body.pdf>

and

<https://journals.plos.org/plosone/s/file?id=ba62/PLOSOne_formatting_sample_title_authors_affiliations.pdf>

We have used the above links to review the journal’s style guidelines and edited the manuscript accordingly.

2. When completing the data availability statement of the submission form, you indicated that you will make your data available on acceptance. We strongly recommend all authors decide on a data sharing plan before acceptance, as the process can be lengthy and hold up publication timelines. Please note that, though access restrictions are acceptable now, your entire data will need to be made freely accessible if your manuscript is accepted for publication. This policy applies to all data except where public deposition would breach compliance with the protocol approved by your research ethics board. If you are unable to adhere to our open data policy, please kindly revise your statement to explain your reasoning and we will seek the editor's input on an exemption. Please be assured that, once you have provided your new statement, the assessment of your exemption will not hold up the peer review process.

We have no objections to the policies of the journal. All data has been uploaded to SRA and GenBank in June 2024 and I recently checked the progress of our submission and it is still listed as processing. We have changed our response to the data availability statement at re-submission.

Additional Editor Comments:

This article is well-suited for publication, and the authors have made a commendable effort in writing and presenting the conclusions. However, some revisions are necessary before it can be published. Please address the reviewer comments and queries, and make the necessary corrections and modifications

Reviewer's Responses to Questions

**Comments to the Author**

1. Is the manuscript technically sound, and do the data support the conclusions?

The manuscript must describe a technically sound piece of scientific research with data that supports the conclusions. Experiments must have been conducted rigorously, with appropriate controls, replication, and sample sizes. The conclusions must be drawn appropriately based on the data presented.

Reviewer #1: Partly

Reviewer #2: Yes

Reviewer #3: Yes

3. Have the authors made all data underlying the findings in their manuscript fully available?

The [PLOS Data policy](http://c05y1x9s.r.us-east-2.awstrack.me/L0/http:%2F%2Fwww.plosone.org%2Fstatic%2Fpolicies.action%23sharing/1/010f0191f7ef2ecd-b255faa3-5f5e-4092-8cb4-ba1957c3ade9-000000/NzE7pXTfQWnLWenZVeH9zN7PfSk=176) requires authors to make all data underlying the findings described in their manuscript fully available without restriction, with rare exception (please refer to the Data Availability Statement in the manuscript PDF file). The data should be provided as part of the manuscript or its supporting information, or deposited to a public repository. For example, in addition to summary statistics, the data points behind means, medians and variance measures should be available. If there are restrictions on publicly sharing data—e.g. participant privacy or use of data from a third party—those must be specified.

Reviewer #1: No

Reviewer #2: No

Reviewer #3: Yes

**Reviewer #1:**

The reviewed manuscript aimed to comparative analysis of whole genomes of P*asteurella multocida* strains isolated from companion animals in Australia with those available in public databases. The authors presented very interesting and important study with the strains which were recently characterized by this research group (ref 12 in the present manuscript).

Specific comments:
Line 26 - Pasteurella multocida (Pm) could infect not only animals as the authors cites in the section Introduction (lines 51-53), please, correct;

We have described *Pasteurella multocida* as being zoonotic in the abstract, thus referring to its ability to cause disease humans as well as animals.

Lines 29-30 – healthy pets and other animals? if not, clinical diagnosis should be indicated.

The isolates of *Pasteurella multocida* were cultured from samples submitted to the Clinical Microbiology Laboratory MVS Werribee over a period of 17 years (2006-2023) for investigation. We have included the term clinical in this sentence to clarify these isolates were collected from animals with clinical signs of pasteurellosis.

Alternatively, the reason for the current bacterial investigation must be included. Details of the sites

The information about the archival 59 isolates featured in this study now appears in a separate supplementary table (S1 Table). We have included additional information about the host disease, sample site, subspecies and carriage of the cryptic plasmid (U51470).

Additionally for captive wildlife - are their zoo animals?

Yes, animals listed as captive are housed in zoos. This has also been included In the S1 Table.

Line 36 - novel STs should be listed;

Our intention is to submit the full genomic sequences to the PubMLST database after publication. It takes some time for a new sequence type to be assigned, and we have decided to precede with publication rather than wait. The details of our analyses using both MLST schemes are included in Table 1.

Lines 40-42 – how much? n=….(% from totally investigated Australian P.
multocida isolates);

We have included the percentage (99/289; 34.3%) for the Australian genomes from various hosts where the cryptic plasmid, U51470, was detected.

Line 43 – similarly, the number of isolates with AMR (n=…, %) and the AMR spectrum detected in isolates from Australian companion animals must be added

We have removed the word ‘rarely’ from this sentence. It now reads: “AMR was not detected in the isolates from Australian companion animals and captive wildlife”.

It is not the first time that AMR has been detected in Australian bovine isolates and the numbers in each category are small, thus we think expressing the number of resistant isolates as a percentage would likely overstate these results and overshadow one of our key findings. Instead, we have included the fractions (2/10 bovine; 1/17 avian)

Line 49 – please, include ‘companion animal’, ‘Pasteurella multocida’, ‘ST’, ‘whole genome’ etc. to the keywords to make your research more available for search by worldwide scientific community

We have revised the keywords to include those suggested by the reviewer and zoonotic transmission.

Lines 86-93 – more information on the Pm isolates must be added, i.e., the strains were isolated from healthy animals?

Please see our earlier comment

Any pasteurellosis cases in animals/humans/owners were related/ associated?

These are isolates collected from clinical cases of disease over an 18 year period.

Unfortunately, we are unaware of any human cases of pasteurellosis related to these cases involving animals.

Did any other either bacterial or viral strains were isolated from the same specimens?

The isolates were obtained via a standard clinical bacteriological diagnostic process using culture and conventional biochemical tests. It was deemed *P. multocida* was the aetiology agent of the disease in all cases.

Line 144 - the accession numbers need to be check to make them available for any potential reader;

The meaning of this comment is a little unclear. However, the 59 isolates are stored and can be made available to any other researchers that wish to include them in their research activities.

Which Pm subspp. was finally identified? This information should be clearer indicated through the manuscript including the section Abstract

We have conducted further analyses to determine the *P. multocida* subspecies using an *insilico* approach to replicate the methods described [1]. The results of these analyses are presented in S1 Table and Fig 7.

The legend for Figure 2 needs to include more details in description;

We have edited the legend for Figure 2 to include details of the different data sources and citations for the recent studies.

The program RAMI used previously is no longer available and any reference to this program has been removed. More details about how we adjusted our approach to this analysis is provided in the materials and methods.

alternatively, Figures 2 and 3 could be companied in a single Figure;

The phylogenetic trees presented in Figures 2 and 3 were generated using different methods (Figure 2, neighbor-joining tree using PopPUNK and Figure 3, Maximum likelihood using SNP analysis) and therefore we think they should not be presented in a combined figure.

The similarity between the overall structures of the phylogenetic tree provides confirmatory evidence the structure of the phylogeny is sound. We would prefer to include both figures in the main document, however Figure 2 could potentially be included in the Supplementary material on the advice from the editor.

We have edited the first paragraph of the section entitled, “Australian isolated have widely diverse genotypes...” to improve the explanation for the purposes for conducting both types of analyses.

Lines 235-238 – the concatenated sequences for housekeeping genes should be deposited in PubMLST database to determine the relevant ST for each of the Australian isolates with unclassified STs

Please see our earlier comments

The data should be added to the Table 1; the alleles with mutation could be highlighted to make these data more visible for potential readers;

Footnotes in Table 1 currently denote the alleles with mutations.

The data presented in the section ‘Phylogeny and MLST strongly suggest multiple instances of cross-species transmission in companion animals’ needs to more evidences to make the conclusions concerning a cross-species transmission of Pm from companion animals to other host spp.

Upon the advice of reviewer 2, we have included recently published Australian *P. multocida* genomic sequences from humans and cats/dogs [2] and used the program PHYLOViZ goeBURST to explore the relationships between 585 *P. multocida* genomic sequences. We were able to identify 5 clonal complexes (presented Table 2 and Fig 5) and pairwise SNP analysis by the program snippy (Table 3) confirms some genomes from different hosts have fewer than 100 SNPs, indicating they are closely related. With inclusion of the more recent genomes from Australia, we have observed more examples of putative cross-species transmissions.

No information was presented on clinical cases in owners & zoo/farm personnel & closely living or contacting wild/domestic animals etc. and companions with Pm-related infection following identification of the identical Pm isolates & genomes.

This is a retrospective study where archival isolates, cultured from clinical specimens submitted to a diagnostic laboratory over a 17 year period, were sequenced. Unfortunately, the types of information suggested by the reviewer are not available. In the discussion, we now state that cross-species, and potentially zoonotic, pasteurellosis in Australian pets, production animals and humans requires further investigation.

Similar Pm strains are isolated from different hosts being adapted for different hosts. However, no information on either direct or indirect cross-species transmission, i.e. transmission the Pm strain from one host to another;

Questions concerning the direction and how cross-species transmission events occur is beyond the scope of this paper. Like the reviewer, we see answers to these questions as being important to improving the management and control of pasteurellosis. In the discussion, we have suggested possible avenues for future research, such as investigating carriage of *P. multocida* by predatory animals, important in the Australian context (ie: foxes, feral cats and rodents).

Line 298 – please, indicate in footnote the source for the information presented in Table 3;

PLASMe was the tool used to detect the presence of plasmids in the sequenced genomes. Rather than add a footnote to Table 3 (now Table 4), as suggested, the sentence in the preceding paragraph has been edited to improve the link between the source of information and Table.

We have also added a column with the heading, Plasmid U51470 to S1 Table to indicate the isolates within this study that we have identified as carrying this plasmid.

Lines 335 through the section AMR – no complete data on AMR for Pm strains investigated could be received, if some genomes were analyzed as contigs;

The 59 genomes were fully circularised using Unicycler, prior to analysis with ABRIcate. We have also conducted phenotypic testing for an array of antimicrobial agents. The reported results from genotypic analysis matched those from the phenotypic tests. Therefore, we consider the information presented accurately reflects the levels of antimicrobial resistance in the 59 new *P. multocida* isolates documented in this study.

the results demonstrated the presence of AMR in the Pm strains should be highlighted to make the data presented in Table 4 more clear for potential readers;

All results above the (T)ECOFF values, provided in the first column, have been coloured red.

Lines 457-458 – P. multocida is very important pathogen for both veterinary and Public Health worldwide. More than 1,000 papers published could be found easily: <https://pubmed.ncbi.nlm.nih.gov/?term=Pasteurella+multocida+AND+human+infection&sort=date>.

We agree with the reviewer that *P. multocida* is an important pathogen.

**Reviewer #2:**

The manuscript presents a genomic and phylogenetic analysis of Pasteurella multocida isolates, with a focus on Australian companion animals. It is a well-written study that significantly contributes to understanding the genomic diversity of this pathogen. However, it overlooks several key publications, particularly recent genomic studies from Australia. While I understand that some of these publications on P. multocida from human and companion animals were released after the authors' NCBI GenBank access date (December 13, 2023), it is unfortunate that they will need to redo portions of their analysis. As their work focuses on Australia, incorporating this new data would strengthen the manuscript's relevance and provide a more comprehensive discussion of the pathogen in this region. Below, I provide a detailed review with recommendations to include the new genomic data, improve clarity, and refine specific analyses.

The manuscript claims there were no genome from Australian companion animals at the date of NCBI GenBank database access. However, genomes of several P. multocida isolates from human and companion animals from Australia have been analysed and deposited in public database. Given the manuscript’s focus on Australian isolates, I recommend incorporating this recent data to update the results and discussion.

We thank the reviewer for alerting us to the recent release of genomic sequence data for *Pasteurella multocida* isolated from cats and dogs and the article comparing these isolates with Australian human isolates.

We note that the sequence type of the feline isolates (PM1613 and PM1620) from the upper respiratory tract is ST20, previously only reported in Australian avian species. In contrast, various sequence types, not including ST20, were isolated from clinical specimens from 2006-2023 in this study, while ST20 continued to be isolated from avian hosts throughout this period.

We have removed all references to there being no complete genomes sequences of *P. multocida* isolated from Australian companion animals available in public databases from the introduction and the first paragraph of the results section.

We have included all fully sequenced Australian genomes isolated from humans, companion animals and a pig. This brings the total number of isolates to 744 in our comparative genome analyses dataset.

Specifically, the following publication available from March 1, 2024 could significantly inform the current analysis:

Smallman TR, Perlaza-Jiménez L, Wang X, Korman TM, Kotsanas D, Gibson JS, et al. Pathogenomic analysis and characterization of Pasteurella multocida strains recovered from human infections. Microbiol Spectr. 2024;12(4):e0380523. (available from 1st of March 2024)

We have cited this reference in the introduction and discussion.

Furthermore, the authors may want to consider incorporating data from a recently published complete genome of a P. multocida isolate from an Australian pig
Chau D, Blackall PJ, Turni C, Omaleki L. Complete genome sequence of a Pasteurella multocida isolate from a pig in Australia. Microbiology resource announcements.0(0):e00241-24.

We have included the genomic sequence data for this isolate (PM1463) in our analyses.

Result section:
Figures 2 and 3: The phylogenetic trees are similar to those described in the Smallman et al. publication, with a distinct deep-branched cluster. However, the quality of the images in this manuscript made it difficult to access the phylogenetic relationships.

We have used the PACE tool to ensure these images comply with the requirements of Plosone.

To improve the clarity and robustness of the phylogenetic analysis, I suggest comparing these results with those from Smallman et al., which may offer additional insights and reinforce the authors' conclusions.

We have repeated our comparative analyses with an expanded dataset set that includes all Australian complete genomes downloaded from Genebank. These include the genomes referenced in Smallman et al. (2024).

Line 255: The manuscript suggests that close phylogenetic relatedness and identical MLST profiles indicate host transfers. While this is possible, it is not definitive. Tools such as PHYLOViZ could be used to more accurately represent evolutionary relationships between strains.

The evidence that we have presented for cross species transfers is also based on pairwise SNP analysis to confirm the close relatedness between isolates, initially observed in the maximum likelihood trees presented in Figs 3 and 4 and MLST profiles (Table 1). The sequences of CM2007-0542-0 and CM2013-0823-1 differ by over 500 SNPs and therefore are not clonal.

We have used PHYLOViZ goeBURST analysis, as suggested by the reviewer, to explore the relationships between isolates. Please see earlier comment raised by Reviewer 1.

With the inclusion of the recent genomes and the different methodology, we were able to provide further evidence for cross species transfers in Table 2 and describe our observations in the following paragraphs.

For example, the chicken isolate (CM2007-0542-0) and duck isolate (CM2013-0823-1) share a common ancestor but this does not necessarily imply direct transfer from one host to the other.

We thank the reviewer for highlighting how this may be misinterpreted by readers of our paper. It was not our intention to suggest direct transfer been the chicken and duck isolates based solely on the results of phylogenetic trees and MLST profiles, rather these analyses may suggest potential occurrences of cross species transmissions. We have revised this sentence in response to the reviewer’s comment.

We have conducted further analyses (PHYLOViZ and snippy) to assess the relationships between different isolates. The details of these analyses are presented in a new figure (Fig. 5) and Tables 2 and 3.

For the example mentioned, CM2007-0542-0 and CM2013-0823-1 place on the same branch of the maximum likelihood phylogenetic tree (Fig 4) and share the same MLST. However, further analyses using the other tools show these isolates do not occur in a clonal complex and they have over 500 SNPs.

Line 260: The manuscripts states that Australian isolates PM1541 and PM1582 belong to ST451. However, according to the original publication, these isolates were identified as ST274 (both based on RIRDC scheme). I suggest the authors revisit the data to confirm the correct sequence type.

We have reviewed the MLST designation for isolates PM1541 and PM1582 in the PubMLST database. These isolates appear to have been recently reassigned to new sequence types.

Isolate ID 854 (PM1541) MH MLST ST 209 RIRDC ST451

Isolate ID 1794 (PM1582) MH MLST ST 209 RIRDC ST451

Line 262: The Smallman TR et al., publication also demonstrates the clustering of the USA strain FDAARGOS_384 and an Australian human isolate. Comparing the findings from these two manuscripts would provide further insight into global strain distribution and zoonotic transmission patterns.

We agree. The inclusion of the fully sequenced genomes from humans and companion animals featured in Smallman et al. (2024) has strengthen the evidence for cross-species transmission and provides further insight into global strain distribution and transmission patterns.

Line 317: The observation that subspecies classification in published genomes does not match phylogeny closely is an important point. To explore this further, I recommend an in-silico approach using available genomic data to differentiate between subspecies. Ujvári et al. (2022) provide a framework for identifying key genes associated with Pasteurella multocida subspecies, which could be useful for this reanalysis. This method would enable verification of the GenBank metadata and address potential misclassifications without the need for additional laboratory work.
Ujvári, B., Gantelet, H., Magyar, T., 2022. Development of a multiplex PCR assay for the detection of key genes associated with Pasteurella multocida subspecies. J. Vet. Diagn. Invest. 34, 319-322.

We have reviewed the article suggested by the reviewer, documenting a multiplex PCR approach to assignment of the subspecies. We also note that a modified approach was used by Smallman et al. (2024) using fastANI to distinguish between subsp. septica and multocida/gallida and insilco PCR, using the primers to detect *gatD* (unique to gallida) and portion of the 16sRNA (unique to septica) [1], to assign a subspecies to a set of 498 genomes (including the 84 Australian isolates sequenced in our laboratory).

We have decorated the phylogenetic tree and the output from Fast ANI in Fig 7A & B to indicate the subspecies identified by this process. We have also added a column, with the heading subspecies to Table 1 to display the results for each of the isolates in this project.

I also suggest incorporating findings from Alhamami et al. (2023), which focus on antimicrobial resistance genes (ARG) in P. multocida isolates from Australian feedlot cattle. This would enhance the discussion around ARG in P. multocida originated from various host species.
Alhamami T, Roy Chowdhury P, Venter H, Veltman T, Truswell A, Abraham S, et al. Genomic profiling of Pasteurella multocida isolated from feedlot cases of bovine respiratory disease. Vet Microbiol. 2023;283:109773.

We have referred to this paper in the discussion, with regard to comparison of the levels of antimicrobial resistance identified in isolates from BRD affected cattle with our results for isolates from companion animals and captive wildlife. Unfortunately, we weren’t able to include this genome in our analyses as it is not available via NCBI (GenBank or SRA databases).

The mentioned Illumina reads cannot located in PRJNA965909.

Please see earlier comments regarding data availability.

**Reviewer #3:**

This article is very good for publication, and the authors have put forth their best effort in writing and presenting the conclusion. However, some changes are required before its publication. I have mentioned all the points below:
I suggest authors to read and incorporate the information from the following articles and cite them:

mRNA vaccines as an armor to combat the infectious diseases. Travel Medicine and Infectious Disease 52:102550.

Zoonotic diseases in a changing climate scenario: Revisiting the interplay between environmental variables and infectious disease dynamics, Travel Medicine and Infectious Disease, 58:102694.

Nanovaccines: A game changing approach in the fight against infectious diseases. Biomedicine & Pharmacotherapy 167(2023):115597

We have considered the advice provided by this reviewer and decided not to make reference to the 3 suggested articles, as they do not relate to the topic of this manuscript.

1.	Ujvári B, Gantelet H, Magyar T. Development of a multiplex PCR assay for the detection of key genes associated with Pasteurella multocida subspecies. J Vet Diagn Invest. 2022;34(2):319-22. Epub 20211202. doi: 10.1177/10406387211063438.

2.	Smallman TR, Perlaza-Jimenez L, Wang X, Korman TM, Kotsanas D, Gibson JS, et al. Pathogenomic analysis and characterization of Pasteurella multocida strains recovered from human infections. Microbiol Spectr. 2024;12(4):e0380523. Epub 20240301. doi: 10.1128/spectrum.03805-23.
