# Supplementary material for: Comparative genome analysis of Pasteurella multocida from Australian domestic animals suggests broad patterns of transmissions across multiple hosts and origins
Source: PLoS One. 2025 Aug 6;20(8):e0329807. doi: 10.1371/journal.pone.0329807 (PMC12327604; doi:10.1371/journal.pone.0329807)
Supplement: Attachment — Submitted filename: Allen et all Plos One 2024 Review 2 .docx [file pone.0329807.s009.docx]

Thanks to the authors for taking my suggestion to consideration. Please see below minor comments to be addressed before publication.

Fig 1. I can not find ST 20 on the x axis. Based on the manuscript, ST 20 consists of a unique population only found in Australia.

Fig 4. There seems to be a disagreement between the Fig legend in regards to the color strip. From left, they are host category, capsule type and LPS type.

Fig 5, Table 2 and the relevant text can benefit from a little more explanation for making it clear to the reader. First, the authors have not mentioned how many groups they recognised in their phyloviz analysis. Assuming the goeBURST A-E in Table 2 is referring to that grouping, groups C, D and E are not marked on Fig 5. Maybe adding a footnote to Table 2 mentioning that the Chestnut_Teal and Swan isolates are depicted by yellow circle in the Phyloviz analysis makes it easier for the reader to follow. For other groups, I can not find any way for the reader to locate those isolates on Fig 5 and one should simply deal with that.
