# Supplementary material for: Comparative genome analysis of Pasteurella multocida from Australian domestic animals suggests broad patterns of transmissions across multiple hosts and origins
Source: PLoS One. 2025 Aug 6;20(8):e0329807. doi: 10.1371/journal.pone.0329807 (PMC12327604; doi:10.1371/journal.pone.0329807)
Supplement: Attachment — Submitted filename: Response_to_reviewers_auresp_2.docx [file pone.0329807.s011.docx]

Dear PLOS ONE Editors,

Thank you for providing us with the opportunity to submit a revised version of our manuscript.

PONE-D-24-33400R1

Comparative genome analysis of *Pasteurella multocida* from Australian domestic animals suggests broad patterns of transmissions across multiple hosts and origins 

Response to Reviewers:

Comments to the Author

1. If the authors have adequately addressed your comments raised in a previous round of review and you feel that this manuscript is now acceptable for publication, you may indicate that here to bypass the “Comments to the Author” section, enter your conflict of interest statement in the “Confidential to Editor” section, and submit your "Accept" recommendation.

Reviewer #1: (No Response)

Reviewer #2: All comments have been addressed

________________________________

2. Is the manuscript technically sound, and do the data support the conclusions?

The manuscript must describe a technically sound piece of scientific research with data that supports the conclusions. Experiments must have been conducted rigorously, with appropriate controls, replication, and sample sizes. The conclusions must be drawn appropriately based on the data presented.

Reviewer #1: Partly

Reviewer #2: Yes

________________________________

3. Has the statistical analysis been performed appropriately and rigorously?

Reviewer #1: N/A

Reviewer #2: N/A

________________________________

4. Have the authors made all data underlying the findings in their manuscript fully available?

The PLOS Data policy<<http://track.editorialmanager.com/CL0/http:%2F%2Fwww.plosone.org%2Fstatic%2Fpolicies.action%23sharing/1/010f0194653aff86-bbfafa89-39f3-4003-95c3-ba1fbea2e2c1-000000/mAncmxSN9vYFIkKpHxf-IRbZUvXUIU3i02dOKgnLIZo=193>> requires authors to make all data underlying the findings described in their manuscript fully available without restriction, with rare exception (please refer to the Data Availability Statement in the manuscript PDF file). The data should be provided as part of the manuscript or its supporting information, or deposited to a public repository. For example, in addition to summary statistics, the data points behind means, medians and variance measures should be available. If there are restrictions on publicly sharing data—e.g. participant privacy or use of data from a third party—those must be specified.

Reviewer #1: No

Reviewer #2: Yes

________________________________

5. Is the manuscript presented in an intelligible fashion and written in standard English?

PLOS ONE does not copyedit accepted manuscripts, so the language in submitted articles must be clear, correct, and unambiguous. Any typographical or grammatical errors should be corrected at revision, so please note any specific errors here.

Reviewer #1: Yes

Reviewer #2: Yes

________________________________

6. Review Comments to the Author

Please use the space provided to explain your answers to the questions above. You may also include additional comments for the author, including concerns about dual publication, research ethics, or publication ethics. (Please upload your review as an attachment if it exceeds 20,000 characters)

Reviewer #1: The authors of the reviewed manuscript made the majority necessary corrections while they must be sure that all the data presented here are deposited in PubMLST, NCBI and other public repository. First of all, ST-related nucleotide sequences should be mandatory deposited in PubMSLT database and available for potential readers of the Journal according to the general policy of the Plos One (see the answer to my relevant recommendations: 'Our intention is to submit the full genomic sequences to the PubMLST database after publication. It takes some time for a new sequence type to be assigned, and we have decided to precede with publication rather than wait.'). Currently, all data underlying the findings in their manuscript could not considered as ‘fully available’. No additional comments.

We have uploaded the genomic sequences of isolates from this study to PubMLST database, with an embargo in place for 6 months.

Reviewer #2: Thanks to the authors for taking my suggestion to consideration. Please see below minor comments to be addressed before publication.

Fig 1. I cannot find ST 20 on the x axis. Based on the manuscript, ST 20 consists of a unique population only found in Australia.

The Fig. 1 shows only the clonal complexes (not the individual STs), as mentioned in the legend, which are identified explicitly in the PubMLST database. The ST20 was not included in a specific clonal complex at the time of analysis and is therefore not represented in Fig. 1.

Fig 4. There seems to be a disagreement between the Fig legend in regards to the color strip. From left, they are host category, capsule type and LPS type.

The figure legend has been edited.

Fig 5, Table 2 and the relevant text can benefit from a little more explanation for making it clear to the reader. First, the authors have not mentioned how many groups they recognised in their phyloviz analysis.

We have included the information that 76 groups of isolates were identified using PhyloViz.

Assuming the goeBURST A-E in Table 2 is referring to that grouping, groups C, D and E are not marked on Fig 5. Maybe adding a footnote to Table 2 mentioning that the Chestnut_Teal and Swan isolates are depicted by yellow circle in the Phyloviz analysis makes it easier for the reader to follow.

For other groups, I cannot find any way for the reader to locate those isolates on Fig 5 and one should simply deal with that.

The aim of Figure 5 is to depict a broader view of the main groups that are reminiscent of the earlier ML phylogenomics analyses. (Inset B highlights the isolates within the deep branched clade that contains most of the isolates from this study.)

To add the same level of information for all of the goeBURST groups listed in Table 2 would likely make this figure very busy. Therefore, we have decided to only refer to goeBURST A in Figure 5 (shown in Inset A) as we have specifically mentioned this group as being part of a larger interrelated group later in the text.

Table 2 lists the details of the goeBURST groups relating to the 5 clonal complexes (groups of isolates with 0 to 4 loci differences) containing mixed host species to demonstrate the evidence of cross species transmission. We think this table can be viewed as a standalone element without the need to refer to Figure 5.

Based on Reviewer 2’s comments, we acknowledge that the output from the PhyloViz analysis was not clearly described. We have edited the relevant paragraphs and added descriptions of insets A & B to the caption of Fig 5 that clarifies what we intended to say.
