# Supplementary material for: Comparative genome analysis of Pasteurella multocida from Australian domestic animals suggests broad patterns of transmissions across multiple hosts and origins
Source: PLoS One. 2025 Aug 6;20(8):e0329807. doi: 10.1371/journal.pone.0329807 (PMC12327604; doi:10.1371/journal.pone.0329807)
Supplement: Attachment — Submitted filename: Response to reviewers R3.docx [file pone.0329807.s012.docx]

Dear PLOS ONE Editors,

Thank you for providing us with the opportunity to submit a revised version of our manuscript.

PONE-D-24-33400R2

Comparative genome analysis of *Pasteurella multocida* from Australian domestic animals suggests broad patterns of transmissions across multiple hosts and origins  

Journal Requirements:

Please review your reference list to ensure that it is complete and correct. If you have cited papers that have been retracted, please include the rationale for doing so in the manuscript text, or remove these references and replace them with relevant current references. Any changes to the reference list should be mentioned in the rebuttal letter that accompanies your revised manuscript. If you need to cite a retracted article, indicate the article’s retracted status in the References list and also include a citation and full reference for the retraction notice.

Additional Editor Comments:

The authors ignore Reviewer 1's request to make fully available the sequences described in their manuscript. First of all, ST-related nucleotide sequences should be mandatorily deposited in the PubMSLT database and available for potential readers of the Journal according to the general policy of PLOS One.

We did try to fulfill Reviewer #1’s request to make all sequence data available for the review process. We agree with the policies of Plos One in fostering open science by making it a condition for publication that all sequence data must be deposited in relevant databases.

The 59 whole genome sequences were uploaded to pubMLST in January 2025 (see email copy below). All ST loci used to analyse the new genomic sequences were extracted directly from this dataset. They are currently embargoed on the pubMLST server, pending publication of the present manuscript.

We chose not to release the WGS data via pubMLST during the peer-review process, because we are concerned that some of our competitors could use the data without acknowledging our work.

Several revisions are required, including structure enhancements, clarity, and scientific rigor.


Reviewers' comments:

Reviewer's Responses to Questions

**Comments to the Author**

1. If the authors have adequately addressed your comments raised in a previous round of review and you feel that this manuscript is now acceptable for publication, you may indicate that here to bypass the “Comments to the Author” section, enter your conflict of interest statement in the “Confidential to Editor” section, and submit your "Accept" recommendation.

Reviewer #1: All comments have been addressed

Reviewer #4: (No Response)

2. Is the manuscript technically sound, and do the data support the conclusions?

The manuscript must describe a technically sound piece of scientific research with data that supports the conclusions. Experiments must have been conducted rigorously, with appropriate controls, replication, and sample sizes. The conclusions must be drawn appropriately based on the data presented.

Reviewer #1: Partly

Reviewer #4: Yes

3. Has the statistical analysis been performed appropriately and rigorously?

Reviewer #1: Yes

Reviewer #4: N/A

4. Have the authors made all data underlying the findings in their manuscript fully available?

The [PLOS Data policy](http://track.editorialmanager.com/CL0/http:%2F%2Fwww.plosone.org%2Fstatic%2Fpolicies.action%23sharing/1/010f0196205fc69f-a458b8da-5088-4fe8-b165-9ec3b71d5f37-000000/4skUb9S9Z0Dq6XhNQERze0rKas2gqxWlS5xIrYrPl78=206) requires authors to make all data underlying the findings described in their manuscript fully available without restriction, with rare exception (please refer to the Data Availability Statement in the manuscript PDF file). The data should be provided as part of the manuscript or its supporting information, or deposited to a public repository. For example, in addition to summary statistics, the data points behind means, medians and variance measures should be available. If there are restrictions on publicly sharing data—e.g. participant privacy or use of data from a third party—those must be specified.

Reviewer #1: No

Reviewer #4: Yes

5. Is the manuscript presented in an intelligible fashion and written in standard English?

PLOS ONE does not copyedit accepted manuscripts, so the language in submitted articles must be clear, correct, and unambiguous. Any typographical or grammatical errors should be corrected at revision, so please note any specific errors here.

Reviewer #1: Yes

Reviewer #4: Yes

6. Review Comments to the Author

Please use the space provided to explain your answers to the questions above. You may also include additional comments for the author, including concerns about dual publication, research ethics, or publication ethics. (Please upload your review as an attachment if it exceeds 20,000 characters)

**Reviewer #1:** No additional comments. The authors should read my previous comments. In the current state, current form, not all data is fully accessible to potential readers.

Reviewer #1 Previous comment

The authors of the reviewed manuscript made the majority necessary corrections while they must be sure that all the data presented here are deposited in PubMLST, NCBI and other public repository. First of all, ST-related nucleotide sequences should be mandatory deposited in PubMSLT database and available for potential readers of the Journal according to the general policy of the Plos One (see the answer to my relevant recommendations: 'Our intention is to submit the full genomic sequences to the PubMLST database after publication. It takes some time for a new sequence type to be assigned, and we have decided to precede with publication rather than wait.'). Currently, all data underlying the findings in their manuscript could not considered as ‘fully available’. No additional comments.

 Please refer to our earlier response to a similar comment.

**Reviewer #4:** This paper describes genomic and phylogenetic analyses of Pasteurella multocida strains, focussing on those isolated from Australian companion animals. It supports and extends recent work showing that isolates from domestic animals and human infections are genetically divergent from the better studied P. multocida strains that infect production animals such as chickens, cattle and pigs. This paper highlights likely cross species transmission events by identifying very closely related isolates from different species. This is an important identification, but in my opinion, can be strengthened by clearly identifying the numbers of SNPs that separate isolates in Table 2, better linking Fig. 5 and Table 2, and adding species designations to Table 3 (as outlined in my major comments below). Overall, this paper is well-written and adds significantly to the current understanding of P. multocida phylogeny and host specificity.

Note that I did not review this previously, so the authors have not previously had a chance to address my comments.


1. For the new genome sequences (sequenced in this study) are the assembly statistics available somewhere?

Key assembly statistics are provided in a new supplementary table (S3 Table), which is referred to in the manuscript.

2. Apologies if I am missing something, but I find it difficult to link the Fig. 5 Phyloviz goeBurst analysis and Table 2. Table 2 has GoeBurst groups of A, B, C, D and E but I can’t easily identify where these clusters are in Fig.5. Are these able to be highlighted somehow?

Assuming the goeBURST A-E in Table 2 is referring to that grouping, groups C, D and E are not marked on Fig 5. Maybe adding a footnote to Table 2 mentioning that the Chestnut_Teal and Swan isolates are depicted by yellow circle in the Phyloviz analysis makes it easier for the reader to follow.

For other groups, I cannot find any way for the reader to locate those isolates on Fig 5 and one should simply deal with that.

The main aim of Figure 5 was to depict a broader view of the main groups that are reminiscent of the earlier ML phylogenomics analyses. (Inset B highlights the isolates within the deep branched clade that contains most of the isolates from this study.) We have included a new version of Figure 5 to indicate the isolates placed in each goeBURST group (A-E) defined in Table 2. Adding the entire set of 76 goeBURST groups (any of which contain only 2 or 3 isolates from the same host species) would make the figure hard to read and interpret.

3. Table 2 indicates what are meant to be clonal complexes containing mixed host species. However, none of these appear to be in the Table 3, which shows closest SNP differences. Perhaps SNP differences (compared to the first strain in each group) could be added into Table 2.

Different analysis tools were used to generate the data for Tables 2 & 3.

Table 2 lists the details of the goeBURST groups relating to 5/76 clonal complexes (groups of isolates with 0 to 4 loci differences) found to contain mixed host species. These results provide evidence of cross species transmission.

Table 3 has details of SNP analysis using snippy to conduct pairwise comparisons of assembled genomes assigned to a ST that contains different host species. It is another method that we have used to demonstrate that cross species transmission has potentially occurred. S1 and S2 tables contain the details of the host species and ST for isolates analysed within this study.

The details of the isolates that make up the 76 goeBURST groups has been provided in a new supplementary table (S6 Table). By providing this supplementary information, we do not think it is necessary to add more isolates to Table 3, as suggested by the reviewer.

Was snippy used to compare all NGS reads from one strain against the reference genome of the other strain? Also, were default settings used to call SNPs?

We have edited the information in the materials and method section to clarify how we have used snippy for pairwise comparisons of assembled genomes belonging to STs containing different host species (see Table 3). The program snippy does this by shredding the contigs into 250 bp single-end reads, with uniform coverage. For each pair of genomes considered, one was used as reference (positional argument --ref), the other one as query (positional argument --ctgs). All permutations were used to construct the table. Only SNPs (not ins, del or mnps) were considered and reported in table 3.

While snippy is usually employed to define polymorphisms by aligning Illumina reads to a reference sequence, this tool can also be used to compare whole genomes already assembled from Illumina data. Since the assembled sequences presented in this manuscript have an average depth coverage close to 300x, it effectively this means the SNP calling function of snippy is redundant here. Therefore, we think it is unnecessary to provide information beyond what is described in the edited version of the materials and methods.

4. Table 3 shows closely related strains (by SNP differences) that are likely associated with cross-species transmission. Can host species be added into this table to quickly identify those strains from different species?

We have added footnotes denoting host species to each genome sequence featured in Table 3.

5. I am unclear why you are unwilling to call the deeply separated clade a subspecies septica. The ANI matrix appears to show that clade, which I believe includes the subsp. septica type strain, as all having >98% identity and having less than 98% identity with the other larger multocida/gallicida clade. I think this is very strong evidence for these being septica. I agree that there is no evidence for separation of multocida/gallicida subspecies.

Based on the ANI results, we agree the members of this clade should be assigned to subspecies septica. However, our reasoning for not assigning subspecies septica is the lack of phenotypic test results, in particular fermentation of sorbitol and dulcitol, available for all members of the clade. We have edited the paragraph to clarify our position.

6. For Fig. 7b (the ANI matrix), the legend on the figure has 5 colors but 6 different numerical % values given, so they don’t match up in a standard way. This should probably just have <95.5, 95.5-96.5, 96.5-97.5, 97.5-98.5, 98.5-99.5, >99.5

We have edited the R code used to generate Figure 7b, so that the tick marks accurately correspond to %identity.

1. Line 106; “read length 250x250 bp” is this meant to be 2x250?

The text has been amended.

2. Fig. 2. Even in the high-res version, the small text at 10 o’clock on the outer circles is nearly unreadable. I think it is just repeated in the legend text at bottom left. However, I think it would be better deleted (leaving just the large legend text) or increased in size to make it readable.

The image has been modified and the information is now in the legend.

3. In Fig. 4 P1581 appears twice, once listed as avian and once listed as Pine Siskin.

There are 2 entries for strain P1581 on the NCBI SRA Database, with different metatada in the headers of the corresponding Genbank files. One dataset indicates “avian host” and the other “Pine Siskin”, a bird species. It is likely that the two entries come from the same individual, but we cannot ascertain this, so it is preferable to provide the two assemblies and treat them as separate entities. The details for all isolates were downloaded directly and the details are provided in S2 Table.
