# Supplementary material for: Comparative genome analysis of Pasteurella multocida from Australian domestic animals suggests broad patterns of transmissions across multiple hosts and origins
Source: PLoS One. 2025 Aug 6;20(8):e0329807. doi: 10.1371/journal.pone.0329807 (PMC12327604; doi:10.1371/journal.pone.0329807)
Supplement: Attachment — Submitted filename: Response to reviewers R4.docx [file pone.0329807.s013.docx]

Dear PLOS ONE Editors,

Thank you for providing us with the opportunity to submit a revised version of our manuscript.  

1. If the authors have adequately addressed your comments raised in a previous round of review and you feel that this manuscript is now acceptable for publication, you may indicate that here to bypass the “Comments to the Author” section, enter your conflict of interest statement in the “Confidential to Editor” section, and submit your "Accept" recommendation.

Reviewer #5: (No Response)

Reviewer #6: (No Response)

2. Is the manuscript technically sound, and do the data support the conclusions?

The manuscript must describe a technically sound piece of scientific research with data that supports the conclusions. Experiments must have been conducted rigorously, with appropriate controls, replication, and sample sizes. The conclusions must be drawn appropriately based on the data presented.

Reviewer #5: Yes

Reviewer #6: Yes

3. Has the statistical analysis been performed appropriately and rigorously?

Reviewer #5: N/A

Reviewer #6: Yes

4. Have the authors made all data underlying the findings in their manuscript fully available?

The [PLOS Data policy](http://track.editorialmanager.com/CL0/http:%2F%2Fwww.plosone.org%2Fstatic%2Fpolicies.action%23sharing/1/010f019789c1d5df-cca11f48-05f6-4538-bc36-b7b7652c9bae-000000/aJfsqSnHludn-YMUF6s0-meyWTLz5jrPVD5sog_Q0TI=216) requires authors to make all data underlying the findings described in their manuscript fully available without restriction, with rare exception (please refer to the Data Availability Statement in the manuscript PDF file). The data should be provided as part of the manuscript or its supporting information, or deposited to a public repository. For example, in addition to summary statistics, the data points behind means, medians and variance measures should be available. If there are restrictions on publicly sharing data—e.g. participant privacy or use of data from a third party—those must be specified.

Reviewer #5: Yes

Reviewer #6: Yes

5. Is the manuscript presented in an intelligible fashion and written in standard English?

PLOS ONE does not copyedit accepted manuscripts, so the language in submitted articles must be clear, correct, and unambiguous. Any typographical or grammatical errors should be corrected at revision, so please note any specific errors here.

Reviewer #5: Yes

Reviewer #6: Yes

6. Review Comments to the Author

Please use the space provided to explain your answers to the questions above. You may also include additional comments for the author, including concerns about dual publication, research ethics, or publication ethics. (Please upload your review as an attachment if it exceeds 20,000 characters)

Reviewer #5: After reviewing the original manuscript and the revised versions after earlier reviews, the manuscript has been significantly improved in the areas that were questioned. I have no objections to the revised manuscript.

We thank the reviewer for this comment.

Reviewer #6: This is a well-executed and innovative study that provides important insights into the genomic diversity of Pasteurella multocida in Australian domestic animals, including pets, farm animals, and captive wildlife. The work is highly relevant to the fields of microbiology and molecular epidemiology and stands out by highlighting potential cross-species and zoonotic transmission pathways, which are currently underexplored in the literature.
The comparative genomic approach, the inclusion of underrepresented host species, and the detailed phylogenomic and MLST analyses make this manuscript a valuable contribution to the understanding of P. multocida population structure and dynamics. The detection of clades associated with pets and humans, and the identification of shared sequence types across different hosts, open new perspectives for investigating zoonotic risks.
The manuscript is generally clear, well-written, and well-organized. The figures are informative and support the main findings appropriately.

Minor Suggestions:

1. If available, add more context regarding clinical outcomes or disease manifestations associated with isolates from companion animals and wildlife. The origin of the samples (e.g. respiratory or oral secretion, abscess, urine, wound secretions) should be mentioned, thus helping in the clinical and diagnostic conduct of veterinarians who work in the clinical care of these species. It can be included as a supplementary file (table).

All available information relating to the clinical specimens is detailed in S1 Table.

2. It would be valuable to include a brief section outlining the limitations of the study. For example, challenges in sequencing quality, gaps in metadata (e.g., clinical presentation, host history), limited sample sizes for certain host species, or lack of functional validation for genomic predictions could be acknowledged. This would help contextualize the findings and guide future research directions.

Our study's genomic analysis was robust and comprehensive. We have provided the genome sequencing statistics in Supplementary Table S3, which illustrates the high quality of sequence reads obtained. As detailed in the Materials and Methods section, all assemblies were fully circularised using Unicycler v0.5.0, and FastANI was employed to identify each isolate. Only isolates with >96.5% average nucleotide identity to *P. multocida* to clarify the taxonomic classification of the genomes included in this study. Genome coverage and assembly process did not indicate any specific problems.

We utilised well-established bioinformatics tools to analyse the sequence data. Since our analysis focused on computational predictions and comparisons based on the genomic sequences obtained, the functional validation of these predictions falls beyond the scope of this study.

This study represents a retrospective analysis of archived isolates from diagnostic clinical cases collected over many years. Estimating the prevalence of *P. multocida* in domestic animals was not an objective of this research. Therefore, we do not consider the relatively small sample sizes as a limitation of the study. Instead, the lack of *P. multocida* isolates from domestic animals in other countries, as mentioned in the first point of our discussion, could be viewed as a valuable guide for future research directions. This gap in our current knowledge base presents an opportunity for more comprehensive, geographically diverse sampling in future studies.

3. Please consider including information on animal ethics approval. Even when using clinical or diagnostic samples from animals, ethical oversight by an institutional Animal Ethics Committee is generally required or at least advisable, and mentioning the approval protocol or exemption rationale is important for transparency.

Under Australian law, animal ethics approval is not required for analysis of samples submitted for clinical diagnosis.

4. If ethical approval was not obtained, we suggest that this be acknowledged as a limitation of the study. The lack of an ethics statement might raise questions regarding compliance with international standards for the ethical use of animals in research.

We have included the following statement at the end of the manuscript.

“Animal ethics committee approval was not required for this study as all samples were submitted for veterinary clinical diagnosis.”

5. The discussion attributes the low antimicrobial resistance levels found in the isolates to the controlled and rational use of antimicrobials in Australian livestock, which is indeed commendable. However, an important question that arises is whether these findings could inform antimicrobial therapy choices for *P. multocida* infections in companion or production animals in other countries or regions. Addressing this point could enhance the broader applicability and relevance of the study and further support its alignment with the One Health approach. Strengthening this perspective may increase the impact of the work beyond the Australian context.

Changes have been made to Results section: ‘Australian isolates have low rates of resistance’ to improve the clarity of our interpretation of the phenotypic AST results generated as part of this study.

In the introduction, we highlighted that Australia possesses distinct pathogen populations, a consequence of its geographical isolation and stringent quarantine regulations. This uniqueness is exemplified by the presence of *P. multocida* ST20. Upon comparing the sequences from our study with published sequences from other countries, such as the United States and China, we observed that some sequences from these regions harboured a higher number of antimicrobial resistance genes (ARGs).

Our findings relate to antimicrobial resistance patterns in a relatively small number of archival isolates from various domestic animals, and therefore are unlikely to inform antimicrobial therapeutic choices for companion and production animals outside of Australia. The distinct pathogen populations and potentially different antimicrobial use practices in Australia may result in resistance patterns that are not representative of those in other countries.

This suggestion from this reviewer is interesting, however it is beyond the scope of this paper to comment on the prescribing practices in other countries. Our focus remains on the Australian context and the implications of our findings for local animal health management.

We have also used the phenotypic AST data as a method to validate the output from ABRIcate.

Overall, I strongly support the publication of this article after minor clarifications.

We thank the reviewer for this comment. We believe that these clarifications are now fully integrated in the revised version of our manuscript.
